# Synthesis of Polymer Janus Particles with Tunable Wettability Profiles as Potent Solid Surfactants to Promote Gas Delivery in Aqueous Reaction Media

**DOI:** 10.1021/acsami.1c07259

**Published:** 2021-06-29

**Authors:** Bradley D. Frank, Milena Perovic, Saveh Djalali, Markus Antonietti, Martin Oschatz, Lukas Zeininger

**Affiliations:** † Department of Colloid Chemistry, Max Planck Institute of Colloids and Interfaces, Am Mühlenberg 1, 14476 Potsdam, Germany; ‡ Faculty of Chemistry and Earth Sciences, Friedrich-Schiller-University of Jena, Philosophenweg 7a, 07743 Jena, Germany

**Keywords:** Janus particles, Janus emulsions, catalysis, polymers, self-assembly

## Abstract

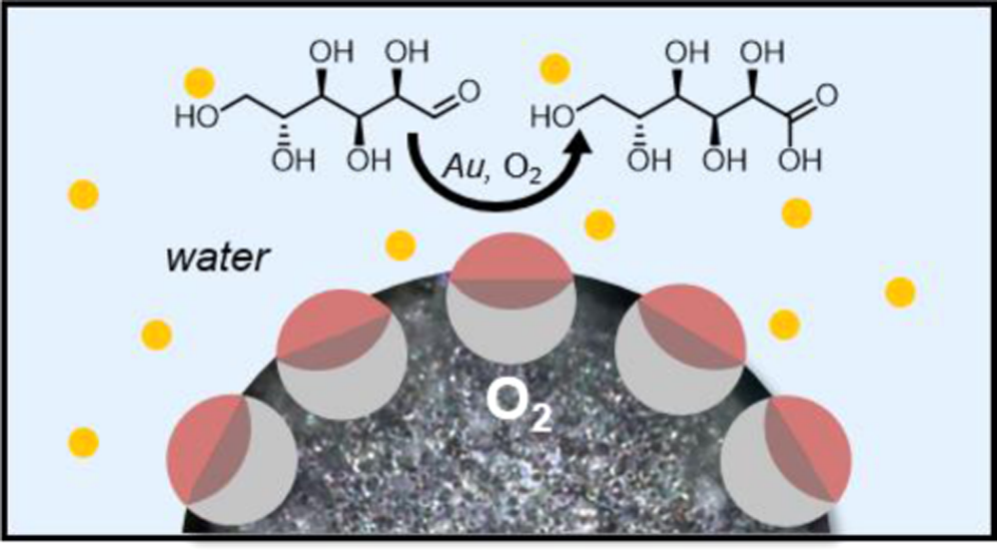

Janus particles exhibit a strong tendency to directionally assemble and segregate to interfaces and thus offer advantages as colloidal analogues of molecular surfactants to improve the stability of multiphasic mixtures. Investigation and application of the unique adsorption properties require synthetic procedures that enable careful design and reliable control over the particles’ asymmetric chemistry and wettability profiles with high morphological uniformity across a sample. Herein, we report on a novel one-step synthetic approach for the generation of amphiphilic polymer Janus particles with highly uniform and tunable wettability contrasts, which is based on using reconfigurable bi-phasic Janus emulsions as versatile particle scaffolds. Two phase-separated acrylate oils were used as the constituent droplet phases and transformed into their solidified Janus particle replicas via UV-induced radical polymerization. Using Janus emulsions as particle precursors offers the advantage that their internal droplet geometry can be fine-tuned by changing the force balance of surface tensions acting at the individual interfaces via surfactants or the volume ratio of the constituent phases. In addition, preassembled functional surfactants at the droplet interfaces can be locked in position upon polymerization, which enables both access toward postfunctionalization reaction schemes and the generation of highly uniform Janus particles with adjustable wettability profiles. Depending on the particle morphology and wettability, their interfacial position can be adjusted, which allows us to stabilize either air bubbles-in-water or water droplets-in-air (liquid marbles). Motivated by the interfacial activity of the particles and particularly the longevity of the resulting particle-stabilized air-in-water bubbles, we explored their ability to promote the delivery of oxygen inside a liquid-phase reaction medium, namely, for the heterogeneous Au-NP-mediated catalytic oxidation of d-glucose. We observed a 2.2-fold increase in the reaction rate attributed to the increase of the local concentration of oxygen around catalysts, thus showcasing a new strategy to overcome the limited solubility of gases in aqueous reaction media.

## 1 Introduction

Janus particles represent a unique collection of functional hybrid materials because they provide asymmetry and can thus impart different chemical or physical properties and directionality within a single-particle system.^1,2^ Due to unique amphiphilic, magnetic, catalytic, optical, or electrical properties, Janus particles can possess advantages over their single-component counterparts with implications for a wide range of applications in the fields of physics, chemistry, and biological science.^3,4^ As such, Janus particles have been used as colloidal building blocks for self-assembled structure formation,^5,6^ in optics and imaging applications,^7,8^ and as motile particles with directional propulsion profiles,^9,10^ transducers and signal amplifiers in sensing applications,^11,12^ and powerful solid surfactants that allow us to control and influence the packing dynamics at interfaces.^13,14^

Amphiphilic Janus particles can mediate superior stability to multiphasic mixtures, such as emulsions, foams, or polymer blends, as opposed to their homogeneous counterparts.^15^ The stability of liquid–liquid or liquid–gas mixtures that are stabilized by particles with a homogeneous surface critically depends on the wettability of the particles. Typically, an intermediate hydrophobicity is most effective and changes in the surface properties can allow for a fine-tuning of the stability, shape, or type of the resulting colloid.^16−18^ However, variations in wettability affect the particles’ free energy of adsorption, which imparts the thermodynamic stability of the colloid against coalescence.^19^ Janus particles, in turn, benefiting from both an increased interfacial adsorption energy of solid particles and their phase-preferential alignment are characterized by an enhanced surface activity.^20−22^ In their most energetically favored orientation, Janus particles localize with each of the two surface domains exposed to their preferred phase, mediating superior stability for multiphase mixtures. This opens the path toward increasing the stability of emulsions, or foams, while allowing for a fine-tuning of interfacial structures and the creation of active, multifunctional surfaces useful for applications, e.g., in imaging, therapy, displays, sensing, and catalysis.^23−27^

To control the interfacial activity of Janus particles, a precise and purpose-defined particle generation method is necessary. While the synthesis of Janus particles with defined structure has been an area of extensive research,^28,29^ the scalable generation of polymer Janus particles with highly uniform surface characteristics still represents a challenge. Traditionally, Janus particles are synthesized using either top-down or bottom-up methodologies. Bottom-up techniques include direct methods, such as self-assembly of functional components, e.g., micelles, co-, or ter-polymers in solution,^30^ and indirect methods, such as a post-surface functionalization of homogeneous particles, e.g., via temporary masking of one particle side using an immobilization template to impart Janus character.^31^ These techniques typically require unique or multistep per-system synthetic approaches and are thus limited in batch scalability. Alternatively, Janus particles can be obtained top-down through polymerization of emulsion droplet molds.^32^ Here, methods range from less precise techniques that give compositional heterogeneity using, e.g., phase separation of polymeric components in emulsions, seeded emulsion polymerization, or controlled and living radical polymerization techniques, to small-volume but more precise microfluidic techniques.^33−35^

Besides generating particles from isotropic emulsion droplets of uniform composition, structured complex multiphase emulsion droplets have been employed as particle templates.^36−39^ The diversity in the anisotropic particle shapes, compositions, sizes, and geometries, is determined by the various controllable internal geometries of the complex droplets and many of these techniques rely on the ability to configure and optimize distinct droplet morphologies prior to emulsification.^40^ As a result, the combination of oils, surfactants, flow conditions, and the use of batch or microfluidic techniques for droplet generation determines the intricacy, shape uniformity, monodispersity, and phase complexity of accessible particle systems.

More recently, Zarzar, Swager, and co-workers reported on a novel thermal phase separation technique for the large-scale generation of shape-uniform emulsion droplets with highly controllable and reconfigurable droplet morphologies.^41^ The mechanism is based on the emulsification of a mixture of two or more fluids above or below their critical solution temperature, where they are miscible. Phase separation after emulsification then yields structured complex emulsion droplets with internal morphologies that reflect the force balance of surface tensions acting at the individual interfaces. In principle, the fabrication technique is applicable to many fluid combinations, including monomer–oil combinations, and applicable to a broad range of emulsification techniques, which determine the size and size dispersity of accessible droplets.^41−43^ The generated droplets are intrinsically responsive and triggerable, i.e., variations in the interfacial tensions evoked by changes in the surfactant type, composition, or effectiveness allow for a fine adjustment of droplet shapes also after droplet generation.^44^ Thus far, this stimuli-responsive nature of the droplets has been exploited in a number of applications, including as tunable micro-optical components^45^ or as biochemical sensors.^8,46,47^

The potential to bulk-generate Janus droplets in highly uniform and reconfigurable internal morphologies offers an attractive platform as broadly accessible particle precursors in the context of the generating functional, spherical, and amphiphilic solid polymer Janus particles. In the herein reported approach, we make use of the temperature-dependent miscibility of two acrylate oils to form the constituent droplet phases of reconfigurable bi-phasic Janus emulsions. Upon formation of the emulsion droplets, UV-induced radical polymerization readily transformed the Janus emulsions into their solidified Janus particle replicas. The structure of Janus emulsion precursors and thus the properties of the final solidified Janus particles could be fine-tuned by adjustment of the surfactant ratio in the aqueous continuous phase, the volume ratio of dispersed phase, and by admixing functional surfactants. Particles generated using this method displayed a strong wettability contrast between the two sides of the particles, which proved useful for a pronounced stabilization of air bubbles-in-water. The increased stability and longevity of the gas bubbles inside an aqueous continuous phase helped to significantly increase the gas content within an aqueous reaction medium and thus improved the performance of a liquid-phase oxidation reaction.

## 2 Experimental Section

### 2.1 Materials

All chemicals were used as received unless stated otherwise: Zonyl FS-300 (40% solids in water, ABCR), sodium dodecyl sulfate (99%, Sigma-Aldrich), 1*H*,1*H*,2*H*,2*H*-perfluorodecyl acrylate (97%, ABCR), 1,6 hexanediol diacrylate (80%, Sigma-Aldrich), 2-hydroxy-2-methyl-propiophenone (97%, Sigma-Aldrich), 4-dimethylaminopyridin (99%, Sigma-Aldrich), *N*-(3-dimethylaminopropyl)-*N*′-ethylcarbodiimide hydrochloride (98%, Sigma-Aldrich), 1-hydroxybenzotriazole hydrate (99%, Sigma-Aldrich), fluoresceinamine (Sigma-Aldrich), 1-octanol (99%, Sigma), gold(III) chloride trihydrate (49 wt % Au Sigma-Aldrich), sodium citrate, sodium borohydride, 10-hydroxydecanoic acid (technical grade, Sigma-Aldrich), trimethylamine (99%, Sigma-Aldrich), acryloyl chloride (97%, Sigma-Aldrich), dichloromethane (Sigma-Aldrich), Pluronic F-127 (Sigma-Aldrich), dioctyl sulfosuccinate sodium salt (Sigma-Aldrich), trimethylolpropane ethoxylate triacrylate (∼428 Mn, Sigma-Aldrich), and d-glucose (Carl Roth). Deionized water was used as the emulsion continuous phase. 10-(Acryloyloxy)decanoic acid (ADA) was synthesized using a modified literature procedure (see the Supporting Information for details).^49^ Gold nanoparticles were freshly prepared according to a known literature procedure.^50^

### 2.2 General Procedure for the Generation of Complex Emulsion Particle Precursors

Bi-phasic Janus emulsion droplets were prepared using a one-step thermal phase separation method.^41,42^ The oil phase consisted of a 1:1 mixture of the acrylate oils 1,6 hexanediol diacrylate (HDDA) and perfluorodecyl acrylate (PFDA). For the purpose of generating of Janus particles with an increased wettability contrast, 6 mg mL^–1^ 10-(acryloyloxy)decanoic acid (ADA) was added to the dispersed phase prior to emulsification. The monomer mixture exhibits an upper critical solution temperature of *T*_C_ = 18 °C, and above this temperature, they form a homogeneous mixture, which was emulsified within an aqueous continuous phase containing different ratios of the hydrocarbon and fluorocarbon surfactants dioctyl sulfosuccinate sodium salt (AOT) and Zonyl FS-300 (Zonyl), respectively. In a typical experiment, 100 μL of the dispersed phase was quickly added to the aqueous surfactant phase (1 mL), before emulsification via vortex mixing for 10 s at 2500 rpm (Digital Vortex-Genie 2). Smaller droplets were obtained via vortex mixing (20 s at 2800 rpm) or bath sonication (bath sonicator, VWR USC-TH; 180 W; 45 kHz; 5 min). The resulting homogeneously mixed droplets were cooled below *T*_C_ by placing the emulsions in a water–ice bath for 2 h to ensure phase separation.

### 2.3 Generation of Complex Emulsion Templated Janus Particles

Phase-separated complex emulsions containing the radical initiator 2-hydroxy-2-methylpropiophenone (Darocur 1173, D1173) in the dispersed phase (4 wt %) were kept inside a water–ice bath and placed under a UV lamp (50 W). Upon polymerization for 15 min, the resulting particles were filtered off and rinsed three times each with water and methanol to remove excess of surfactant and then dried under air before imaging.

### 2.4 Generation of Volume-Ratio-Tuned Particles with Varied Amphiphilicity and ECA Treatment

Further particle tuning was accessed via volume ratio. For this, complex droplet templates were prepared with a solution of 6 mg mL^–1^ ADA in 1,6 hexanediol diacrylate with 5 wt % trimethylolpropane ethoxylate triacrylate and 5 wt % darocur 1173 as the hydrocarbon phase (HC) and PFDA as the fluorocarbon phase (FC). To prepare droplets with varying volume ratios, volume ratios (*V*_HC_/*V*_FC_) of 8:2, 7:3, 6:4, 4:6, and 3:7 were made as the dispersed phases. Each dispersed phase was emulsified in a 2:8 dioctyl sulfosuccinate sodium salt (AOT, 1 wt %):Zonyl via vortex mixing at 2500 rpm for 10 s, to generate complex droplets with varying volume ratios but a static contact angle. These emulsions were cooled below *T*_C_ to induce phase separation for 2 h, before polymerization under a UV lamp (50 W) for 15 min. Polymer Janus particles were filtered off and rinsed three times each with water and methanol before drying in air.

### 2.5 Microscopy

Janus particles and complex emulsion shapes and sizes were monitored using an optical microscope (Leica DVM digital microscope) and an inverted microscope (Bresser IVM 401) with the emulsions and particles dispersed in an Invitrogen Attofluor Cell Chamber (Thermo Fisher Scientific). Horizontal imaging was performed using a customized side-view microscopy setup with variable zoom, composed of two tube 200 mm tube lenses, a HIKVision area scan CCD camera, and an Olympus planar optical microscopy lenses, utilizing 100 and 200 μm cuvettes (Hellma Analytics), as well as generic cavity slides. For particle interfacial contact angle and Janus ratio determination, particles were dispersed in a 1 wt % solution of Pluronic F-127 prior to imaging on the side-view optical microscope. Scanning electron microscopy (SEM, 3 kV) was undertaken on a Zeiss Leo Gemini 1550 instrument. Confocal microscopy was performed on a Leica (Leica SP8). Average particle diameters of small (∼1 μm) particles were determined using dynamic light scattering (DLS; Malvern Panalytica Zetasizer Nano).

### 2.6 Side-Selective Postfunctionalization of Polymer Janus Particles

Amphiphilic particles generated with ADA organized to the hydrocarbon–water interface on polymerization were dispersed in 4 mL of DMF over ice. Subsequently, 4-dimethylaminopyridin (10 mM) and 1-hydroxybenzotriazole hydrate (10 mM) were added and the dispersion was stirred for 15 min, before *N*-(3-dimethylaminopropyl)-*N*′-ethylcarbodiimide hydrochloride (20 mM) was added. After an additional 15 min, fluoresceinamine (50 mM) was added and the stirred mixture was left overnight at room temperature. To isolate postfunctionalized particles, the particles were filtered off and rinsed three times each with water and methanol and dried under air before imaging.

### 2.7 Determination of Interfacial Contact Angles

Dry particles (powder) were placed at an air–water interface in a disposable vessel. These vessels were placed on a hot plate (60 °C) with insulation from direct heat. Low-viscosity ethyl 2-cyanoacrylate (ECA, 1 mL)-based superglue was placed in an aluminum boat next to the particle-containing vessels with direct contact to the hot plate. Target samples and ECA were covered with a glass container for 1 h on the hot plate, after which the ECA-decorated particles were removed and investigated using SEM. Images were processed with Fiji to calculate the interfacial water contact angles. An expanded schematic for ECA treatment is displayed in Figure S9.

### 2.8 Measurement of Oxygen Dissolution Kinetics

Quantification of the oxygen content inside an aqueous phase was undertaken with an optical oxygen meter (PreSens Fibox 3). To measure the influence of the solid surfactant on oxygen delivery, a control measurement of ambient oxygen dissolution into 50 mL of DI water was performed in a three-neck flask after deaeration of the solution via nitrogen bubbling. At a reading of 0 μmol L^–1^, the flask was opened to ambient air and the rate of oxygenation of the solution was measured with the water under constant stirring. To measure the benefit of the solid surfactant to oxygenation, 4 mg mL^–1^ of synthesized and cleaned Janus particles (50 μm diameter, interfacial contact angle of θ = 61°) were added, and the entire system was deoxygenated with nitrogen bubbling until 0 μmol L^–1^ was measured, at which point the flask was opened to ambient air under constant stirring and the oxygen content of the solution was monitored over time.

### 2.9 Catalysis of d-Glucose to Gluconic Acid

Gold-mediated catalysis of d-glucose was undertaken in a three-neck round-bottom flask equipped with a pH meter, condenser, gas inlet, and titrating syringe. First, Janus particles (4 mg L^–1^) were added to 40 mL of a d-glucose solution (0.5 M). Subsequently, we added 10 mL of a gold nanoparticle dispersion (58 mg L^–1^) to start the reaction. The pH value of the reaction solution was maintained at pH = 9 by titration with 1 M sodium hydroxide. The progress of the catalytic reaction was monitored by tracking the added volume of sodium hydroxide with a TitroLine 6000/7000 titration device equipped with a TITRONIC piston burette, and the conversion of d-glucose into gluconic acid was verified via NMR (Supporting Information). All catalysis experiments were undertaken with constant stirring (1000 rpm) at *T* = 30 °C in identically sized flasks. Molecular oxygen was bubbled continuously at 10 mL min^–1^. For the investigation of the Janus particle size dependency of the catalytic reaction rate, the reaction was performed using 45 mL of d-glucose solution (0.5 M) and 5 mL of gold nanoparticle dispersion (58 mg L^–1^).

## 3 Results and Discussion

### 3.1 Synthesis of Amphiphilic Polymer Janus Particles

**Figure 1:**
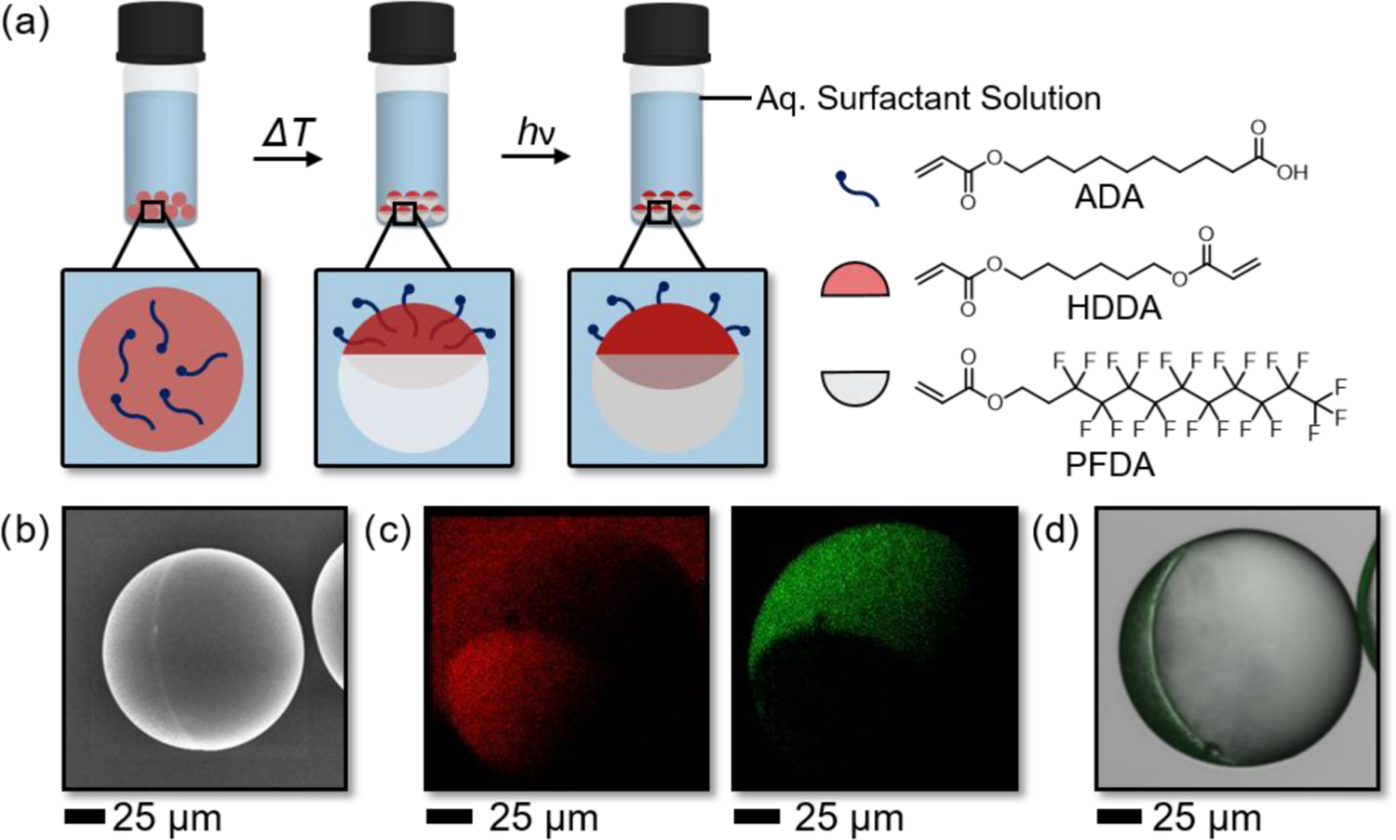
(a) Generation of polymer Janus particles using reconfigurable Janus emulsions as particle molds, where monomer oils phase-separate upon cooling and can subsequently be solidified using UV light. To increase the wettability profile of the particles, an internal carboxy-acrylate surfactant (ADA) was used that selectively assembled to one interface of the emulsions. (b) Scanning electron micrograph of a polymer Janus particle synthesized by this method; scale bar: 25 μm. (c) Energy-dispersive X-ray spectroscopy (EDX) maps indicating the anisotropic distribution of the carbon (red) and fluorine (green) content within the particles; scale bar: 25 μm. (d) Fluorescence micrograph of a postfunctionalized Janus particle, where fluoresceinamine selectively covalently attached to the carboxylic acid functionalized interface of the particles; scale bar: 25 μm.

Our strategy for the generation of amphiphilic Janus particles was based on utilizing reconfigurable complex Janus emulsions as particle templates. To create Janus particles in various morphologies, we therefore started with the generation of the Janus emulsion molds composed of a 1:1 volume mixture of hydrocarbon and fluorocarbon monomers, namely, 1,6 hexanediol diacrylate (HDDA) and perfluorodecyl acrylate (PFDA). The two oils exhibit an upper critical solution temperature of *T*_C_ = 18 °C, above which they form a homogeneous mixture, which we emulsified within an aqueous continuous phase containing the hydrocarbon and fluorocarbon surfactants dioctyl sulfosuccinate sodium salt (AOT) and Zonyl FS-300 (Zonyl), respectively. Upon cooling, the two oils phase-separate into distinct hemispherical compartments yielding Janus emulsions that exhibit highly uniform internal morphologies across a sample. Due to the low internal interfacial tension between the two internal phases compared to the interfacial tensions between the oils and the continuous phase, the droplets exhibit a close-to-spherical shape with the internal droplet curvature dictated by the balance of surface tensions acting at the outer interfaces.^41,44^ The pristine Janus droplets were then UV-polymerized into their corresponding Janus particle replicas (Figure 1a). To this end, we added the radical photoinitiator (2-hydroxy-2-methylpropiophenone, Darocur 1173) and placed the emulsions under a UV lamp (50 W). Upon polymerization, the shape and size of the Janus droplets were essentially retained, resulting in the generation of morphologically uniform polymer Janus particles. Scanning electron microscopy (SEM) revealed the Janus character of the resulting particles (Figure 1b), and energy-dispersive X-ray spectroscopy (EDX) showed the anisotropic distribution of carbon and fluorine content across the particle (Figure 1c).

Due to the different polymers comprising both hemispheres, the as-synthesized particles displayed an intrinsic amphiphilic character, i.e., an asymmetric wettability profile. To estimate the wettability contrast between the individual hemispheres of the particles, 2D static contact angles of water on surface analogues of the two polymers at both sides of the particles were measured (Table S1). The experiments revealed a strong contrast in hydrophilicity with a measured contact angle of θ = 120° for the PFDA polymer, and θ = 76° on the poly-HDDA surface. To further increase this wettability contrast and tune the hydrophilicity of the hydrocarbon polymer interface, we admixed a tailor-synthesized 10-(acryloyloxy)decanoic acid (ADA) surfactant (6 mg mL^–1^) to the oil mixture, which selectively partitioned into the hydrocarbon monomer phase of the Janus droplets and selectively adsorbed onto the hydrocarbon–water interface of the emulsions. Thus, upon co-polymerization with the hydrocarbon polymer under UV light, only one side of the Janus particles was functionalized with carboxylic acid moieties. On surface analogues, these carboxylate functionalities sufficed to lower the static contact angles of water down to θ = 56°. As proof of the carboxylic acids to be present at only one hemisphere of the particles, we performed a postfunctionalization reaction. To this end, we coupled the fluorescent marker fluoresceinamine to the polymerized carboxylic acid functional groups in a 1-ethyl-3-(3-dimethylaminopropyl)carbodiimide (EDC)-mediated coupling reaction. Bulk reaction of the particles with the fluorescent marker yielded particles with the dye functionality present selectively at only one side of the particles, as evidenced by fluorescence microscopy displayed in Figure 1d.

### 3.2 Tuning of Particle Morphology

**Figure 2:**
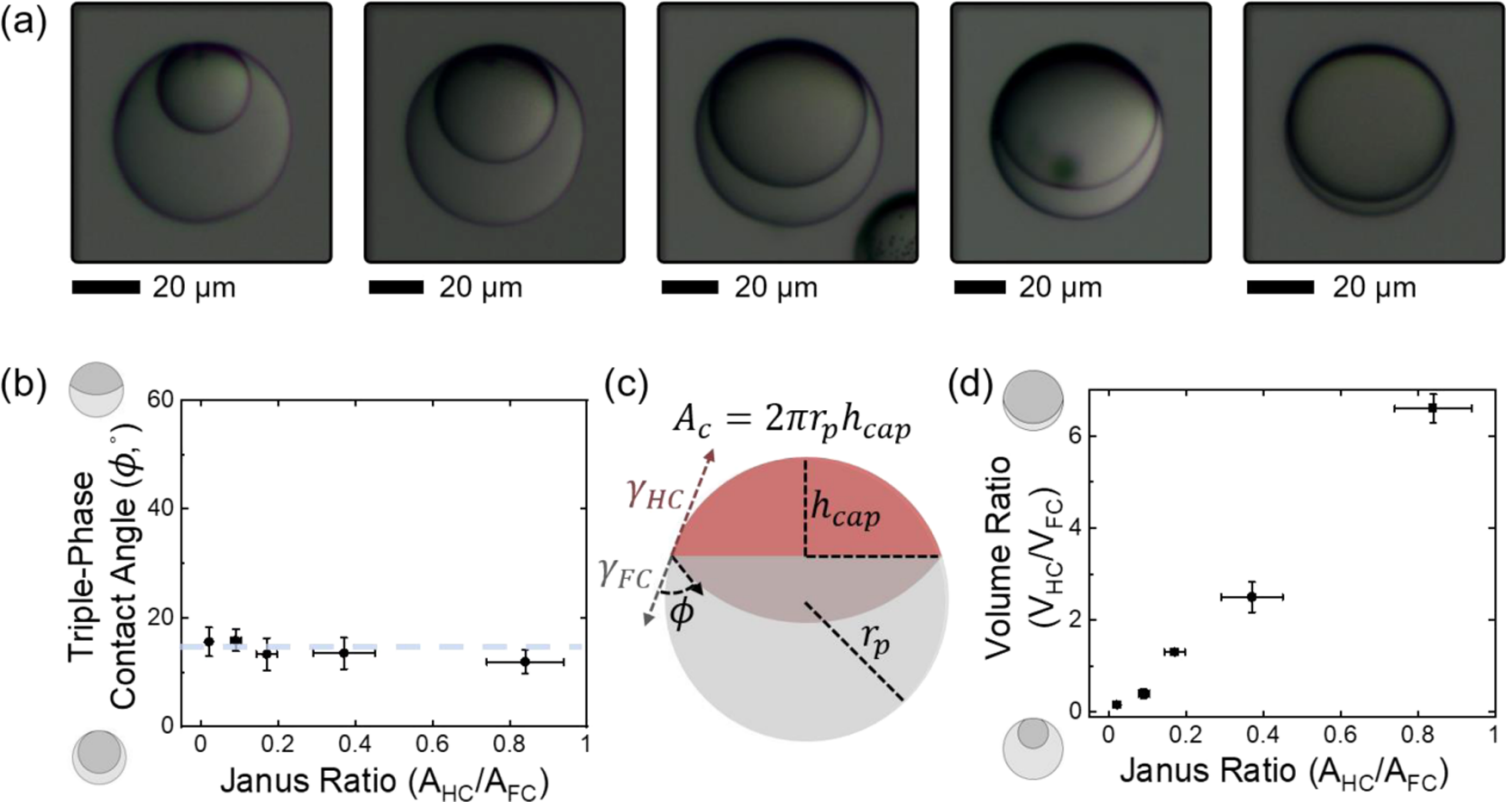
(a) Side-view micrographs of polymer Janus particles synthesized using different starting volume ratios of hydrocarbon and fluorocarbon monomer oils (HC/FC ratio of 3:7, 4:6, 6:4, 7:3, 8:2); scale bar: 20 μm. (b) Particle contact angles reflect the balance of interfacial tensions acting at the individual interfaces and remained constant independent of the employed volume ratio of oils. (c) Schematic representation of the calculation of particle contact angles and the ratio of hydrophilic to hydrophobic surface areas, i.e., the particle Janus ratio. (d) Plot of the ratio of surface areas (Janus ratio) vs the applied volume ratio of monomers showing that the particle wettability profile can be tuned with changes in the particle composition.

The geometry of the resulting spherical Janus particles could be modified as a function of the continuous phase surfactant composition, ADA concentration, as well as the applied volume ratios of the two phases (Figure 2). Changes in the surfactant ratio of AOT and Zonyl allowed variation of the droplet morphology, quantified by determining the triple-phase droplet contact angle Φ between the FC/W and HC/FC interface of the emulsions or formed particles, respectively (Figure S5). In turn, variations in the applied volume ratio of the two monomer components before emulsification yielded changes in the size ratio of the internal compartments (Figure 2a); however, the contact angle at the triple-phase contact line remained constant (Figure 2b). With respect to the wettability contrast of the resulting particle systems, the volume approach enabled a facile and controllable adjustment of the hydrophilic to hydrophobic surface areas. This Janus ratio, which we calculated by determining the ratio of particle surface area of the hydrocarbon spherical cap over the interfacial area covered by the fluorocarbon polymer (Figure 2c; see the Supporting Information for details), changed linearly with volume ratio (Figure 2d).

### 3.3 Interfacial Behavior of Morphology-Tuned Particles

**Figure 3:**
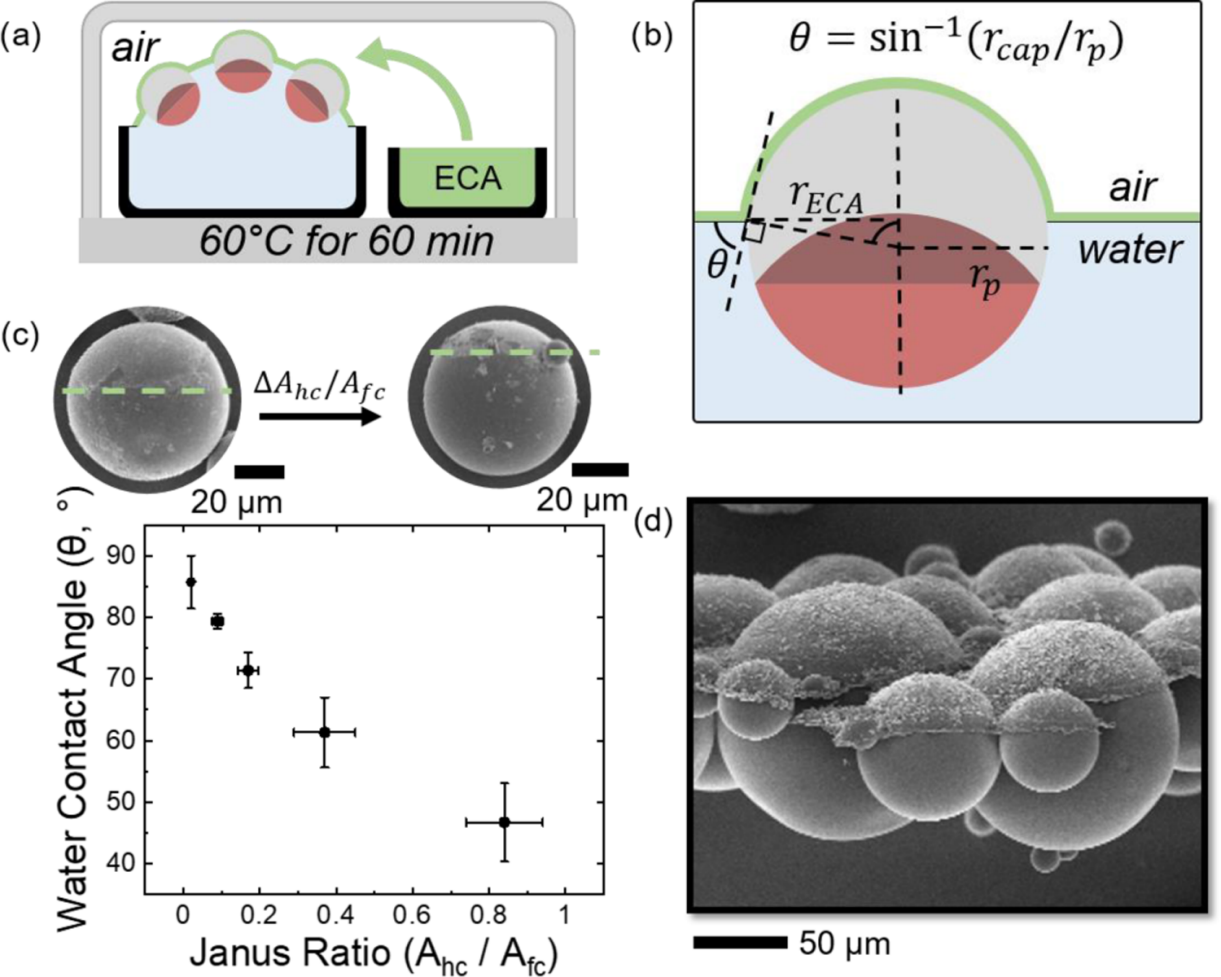
(a) Schematic of an ECA vapor treatment procedure used to determine interfacial contact angles of polymer Janus particles assembled at an air–water interface. (b) Calculation of the interfacial contact angle of polymer Janus particles at an air–water interface. (c) Linear correlation of interfacial contact angles of polymer Janus particles as a function of the ratio of hydrophilic to hydrophobic surface areas (Janus ratio). (d) SEM image of polymer Janus particles after ECA vapor treatment; scale bar: 50 μm.

Having the polymer Janus particles with different wettability contrasts at hand, we next set out to explore their capabilities as solid surfactants. All Janus particles synthesized by this method were characterized by a high tendency to directionally assemble at hydrophilic–hydrophobic interactions, such as air–water interfaces as a result of their amphiphilic character. Janus particles offer advantages as solid surfactants due to their strong tendency to segregate to interfaces with adsorption energies up to three times larger than their homogeneous counterparts, while the wettability contrast allows fine-tuning of the interfacial assembly behavior.^15^ To reveal the differing interfacial assembly behavior of our as-prepared Janus particles with differing Janus ratios, we determined their interfacial contact angles at air–water interfaces. In these experiments, we first deposited the Janus particles at a water–air interface and subsequently solidified these interfaces by a vapor treatment with an ethyl 2-cyanoacrylate-based superglue (ECA) (Figures 3a and S9).^17,48^ SEM images of the coated Janus particles allowed the visualization of their interfacial location and quantification of air–water contact angles, as only air-exposed particle surfaces were exposed to and decorated by the ECA (Figure 3b). The determined interfacial contact angles were observed to be highly consistent across a sample and solely dependent on the particle Janus ratio, independent of their polydisperse size (Figures 3c,d and S9). As a result, tuning the wettability contrast in particles allowed control over their interfacial location and phase preference, thus imparting their potential to stabilize multiphasic mixtures such as emulsions, bubbles, foams, or liquid marbles.

**Figure 4:**
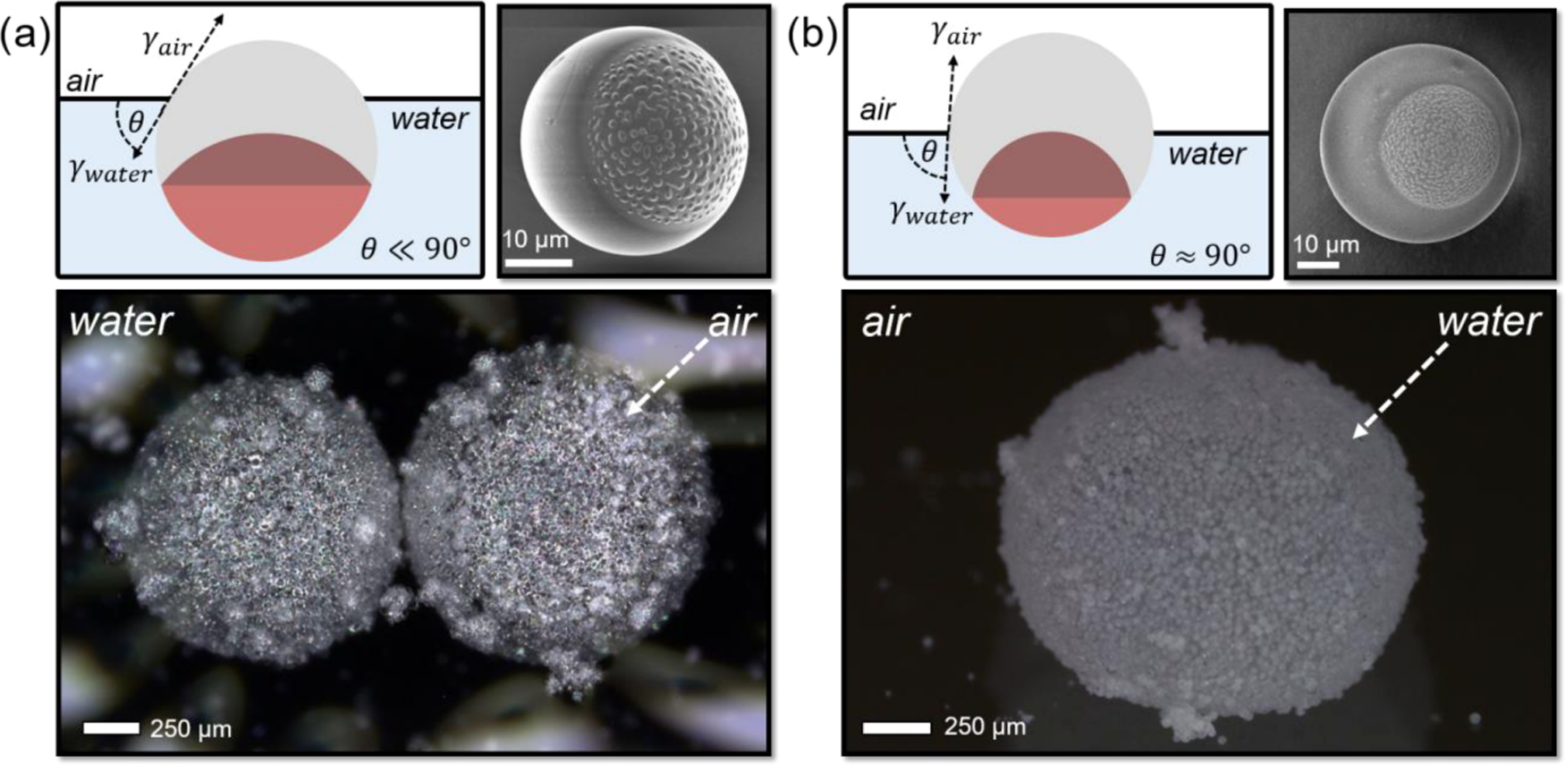
(a) Amphiphilic polymer Janus particles with low interfacial contact angles (SEM images of particles generated with 9 mg mL^–1^ ADA in 1:1 HDDA/PFDA, emulsified in 8:2 AOT/Zonyl (scale bar: 10 μm) preferentially stabilized air-in-water bubbles, such as in the displayed optical micrograph; scale bar: 250 μm). (b) In contrast, synthesized amphiphilic polymer Janus particles with interfacial contact angles close to 90° (SEM images of particles generated with 9 mg mL^–1^ ADA in 1:1 HDDA/PFDA, emulsified in 1:1 AOT/Zonyl (scale bar: 10 μm) could be employed to stabilize a water-in-air liquid marble as shown in the corresponding optical micrograph; scale bar: 250 μm).

With the versatile interfacial activity of the as-prepared polymer Janus particles established, we next set out to utilize the interfacial adhesion potential of the amphiphilic Janus particle to stabilize various air–water interfaces. We started by rolling water droplets on dried particle powders of polymer Janus particles comprising different wettability profiles. In this attempt, only particles with a low Janus ratio, i.e., the particles with a Janus ratio of 0.02 that displayed an interfacial contact angle of θ = 86° were able to successfully stabilize a close to spherical water-in-air liquid marble. In contrast, when vortexing or heavily stirring an aqueous particle dispersion, the more hydrophilic polymer particles, e.g., particles with a Janus ratio of 0.4 that exhibited an interfacial contact angle of θ = 61° (henceforth referred to as the solid surfactant), readily produced stable air-in-water bubbles. Interestingly, these bubbles, once formed, were observed to be highly stable, and depending on their size and particle-to-air ratio, floated in solution or remained beneath the air–water interface for months. These experiments revealed that the interfacial contact angle and thus the phase preference of the polymer Janus particles determined their interfacial stabilization potential as displayed in Figure 4.

### 3.4 Janus Particle-Stabilized Air-in-Water Bubbles to Promote Oxygen Delivery Inside an Aqueous Reaction Medium

**Figure 5:**
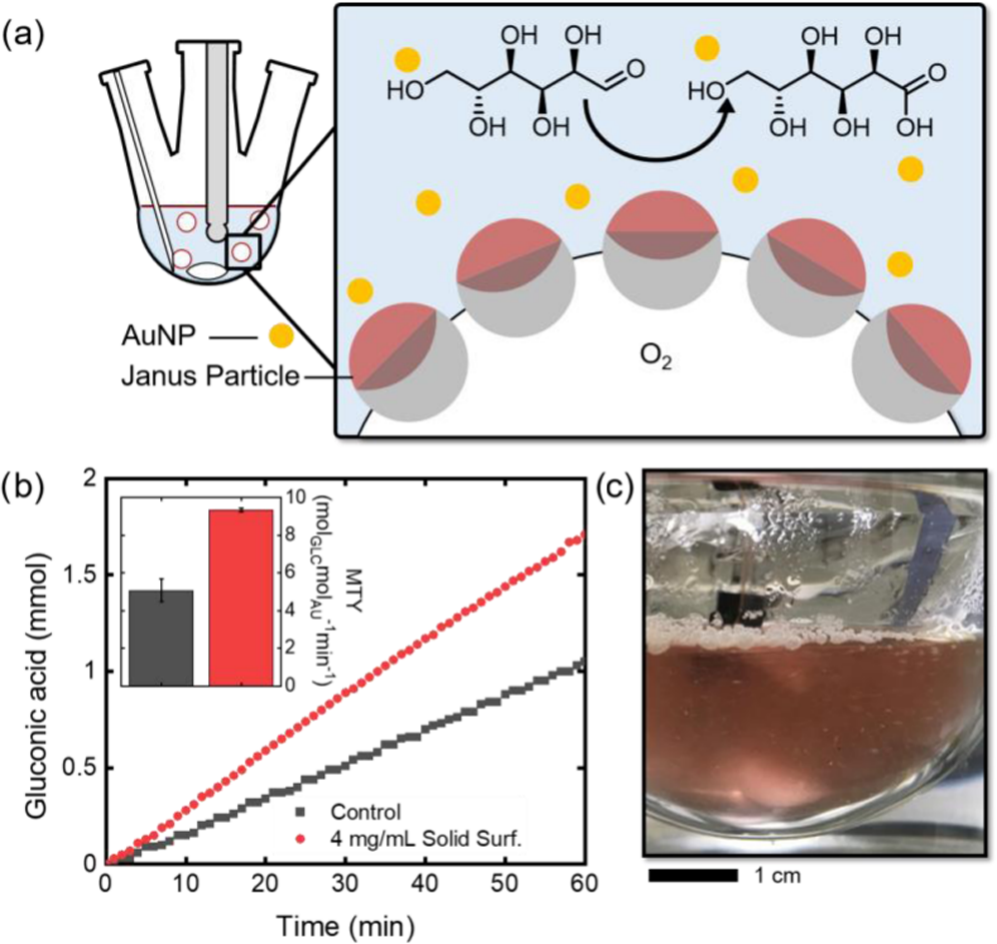
(a) Schematic drawing of the experimental setup for the gold nanoparticle-mediated catalytic oxidation of d-glucose to gluconic acid using molecular oxygen, with an indication of the pH meter, a syringe for oxygen delivery, and the amphiphilic Janus particle-stabilized oxygen bubbles inside the reaction mixture. (b) Reaction monitoring of a gold-mediated conversion of d-glucose to gluconic acid tracked via titration of the reaction medium with 1 M sodium hydroxide with and without addition of amphiphilic Janus particles (4 mg mL^–1^) (reaction conditions: 10 mL min^–1^ O_2_, 0.4 M glucose, 7.36 × 10^–5^ mol_Au_ mol_glc_^–1^ Au nanoparticles, stirring: 1000 rpm, *T* = 30 °C). The inset graph displays the reaction rates expressed in metal time yield (MTY, mol_glc_ mol_Au_^–1^ min^–1^) after a reaction time of 60 min, *n* = 3. (c) Image of reaction solution containing polymer Janus particles displaying the increased cloudiness of the solution; scale bar: 1 cm.

Next, we leveraged the potential of as-synthesized Janus particles (Figure 4a, interfacial contact angle of θ = 61°) that result in low interfacial contact angles to act as solid surfactants for the stabilization of air bubbles in water and tested their ability to increase the gas content and thus promote the delivery of oxygen (Figure S15) inside a liquid-phase oxidation reaction medium. An increase in the local concentrations of gaseous reactants in proximity to the dispersed solid catalysts has been previously associated with an enhanced catalytic performance.^51,52^ We opted for an aqueous phase oxidation reaction of d-glucose to gluconic acid because, apart from being a relevant industrial reaction for producing higher value-added chemicals from biomass, it also presents itself as a defined and well-investigated reaction with high selectivity toward a single product, mild reaction conditions, and simple access to monitor the reaction rate (Figure 5a).^53−55^ In our experiments, we used unsupported gold nanoparticles as catalysts for the aqueous phase d-glucose oxidation that were added as aqueous dispersion (53 mg L^–1^) to an d-glucose solution (0.4 M). Throughout the reaction, the pH value of the mixture was kept constant at pH = 9 via titration with a sodium hydroxide standard solution (1 M). In this scenario, time-dependent monitoring of the added volume of sodium hydroxide can be directly correlated to the d-glucose conversion, which makes this reaction a suitable model reaction for studying the influence of gas delivery toward the active catalytic reaction sites on the reaction rate. As a reference experiment, we first monitored the gold-mediated conversion of d-glucose to gluconic acid under constant bubbling of molecular oxygen (Figure 5b). We determined the metal time yield (MTY), i.e., the amount of glucose converted per mass of gold per time monitored over the linear regime of the titration experiment, of 5.1 mol_glc_ mol_Au_^–1^ min^–1^. The conversion of d-glucose to gluconic acid as the sole reaction product was verified via NMR spectroscopy (see the Supporting Information). However, upon addition of 4 mg mL^–1^ solid surfactant Janus particles to the system, we observed an increase of the MTY of 82% to 9.3 mol_glc_ mol_Au_^–1^ min^–1^, attributed to the increased availability of oxygen inside the aqueous reaction medium. The latter became apparent immediately upon addition of the particles through an increased turbidity of the otherwise transparent reaction medium (Figure 5c). This, in turn, was a result of both the formation of large, stable oxygen bubbles in water, which preferentially floated below the air–water interface, and particularly the generation of smaller assemblies of particles formed via hydrophobic interactions (Figure S10). Considering the hydrophilic/hydrophobic nature of the Janus particles, the solid surfactant assembles entirely into bubbles or microassemblies compared to single-phase counterparts, where hydrophilic or hydrophobic particles disperse easily into solution or prefer to remain at the air–water interface, respectively (Figures S11 and S12). These microassemblies exhibited small volumes of oxygen trapped between the particles and thus present significantly higher particle-to-gas weight ratios, allowing them to freely float in solution.

**Figure 6:**
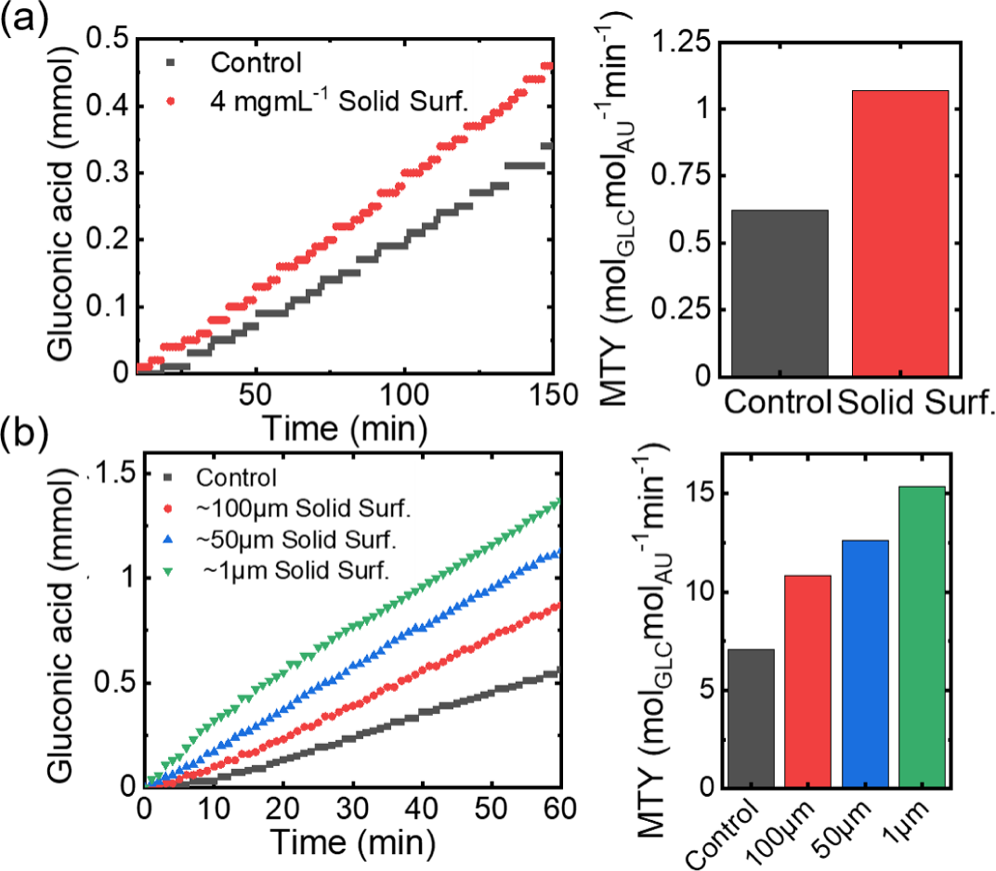
(a) Reference and particle-supported reaction progress and rate for a gold-mediated conversion of d-glucose to gluconic acid without the supply of molecular oxygen (reaction conditions: 0.4 M glucose, 7.36 × 10^–5^ mol_Au_ mol_glc_^–1^ Au nanoparticles, stirring: 1000 rpm, *T* = 30 °C). (b) Particle size dependency of the volume of stabilized oxygen inside the aqueous reaction medium, and thus the reaction performance of the catalytic liquid-phase d-glucose oxidation (reaction conditions: 10 mL min^–1^ O_2_, 0.45 M glucose, 3.27 × 10^–5^ mol_Au_ mol_glc_^–1^ Au nanoparticles, stirring: 1000 rpm, *T* = 30 °C).

In addition, we tested the gold nanoparticle-mediated conversion of d-glucose without bubbling molecular oxygen under ambient conditions, under continuous stirring. In this case, the reaction rate of the reference reaction dropped significantly, yielding an MTY = 0.6 mol_glc_ mol_Au_^–1^ min^–1^. In turn, with the addition of the Janus particle surfactants, an MTY of 1.1 mol_glc_ mol_Au_^–1^ min^–1^ was measured. This improvement of 72% is attributed to the particles’ potential to increase the gas content inside an aqueous reaction medium, as a result of the increased interfacial stabilization of the Janus particles and the oxygen-mediated formation of hydrophobic interaction-driven formation of colloidal self-assembled aggregates in solution (Figures 6a and S10).

Smaller polymeric Janus particles could further increase the gas–liquid interfacial area, thus leading to an increased portion of particle-stabilized air bubbles and colloidal aggregates inside the reaction medium. The general synthetic approach toward polymeric Janus particles via reconfigurable complex emulsions is applicable to many conventional emulsification techniques that allow variation of the resulting particle sizes and size distributions over a wide range. To display a correlation between an increase of the particle surface area with the delivery of oxygen, we tuned the average diameter of the solid Janus particle surfactants in a size range of *d* = 1–100 μm using low- and high-energy methods to generate the emulsion precursors, namely, vortex mixing and sonication (see the Supporting Information for details). With the mass ratio of added solid surfactant held constant at 4 mg mL^–1^, a decrease in particle diameter directly correlated with an increase in the reaction rate, attributed to the increased stabilization of oxygen in the reaction medium due to the increased particle surface area. More specifically, when decreasing the average particle diameter from ∼50 to ∼1 μm, the sizes of the resulting particle-stabilized oxygen bubbles decreased from 301 ± 332 to 23 ± 14 μm, respectively (Figure S13). To monitor the influence of oxygen content inside the reaction medium, we slightly increased the glucose (0.45 M) and lowered the gold nanoparticle (3.27 × 10^–5^ mol_Au_ mol_glc_^–1^) concentrations. Under these conditions, a reference experiment performed without particle addition yielded an MTY of 7.1 mol_glc_ mol_Au_^–1^ min^–1^. Upon addition of larger (*d* ∼ 100 μm) polymer Janus particle surfactants, oxygen delivery was improved, resulting in an MTY of 10.8 mol_glc_ mol_Au_^–1^ min^–1^. However, upon addition of smaller Janus particle surfactants, we observed a significant performance increase in the catalytic oxidation, yielding an MTY of 12.6 mol_glc_ mol_Au_^–1^ min^–1^ using 50 μm particles toward 15.4 mol_glc_ mol_Au_^–1^ min^–1^ using particles with an average size of *d* = 1 μm, respectively (Figure 6b).

## 4 Conclusions

In summary, in this article, we demonstrated a novel approach for the generation of functional spherical polymer Janus particle with tunable amphiphilicities, using reconfigurable bi-phasic Janus emulsions as versatile particle scaffolds. The emulsions were composed of a phase-separated mixture of hydrocarbon- and fluorocarbon-based acrylate monomer oils, and facile control of the internal morphology and composition of the Janus emulsion droplets was realized via tuning the ratio of surfactants, oils, or via admixing of functional components. Specifically, we admixed a tailor-synthesized internal carboxylic acid-acrylate surfactant (ADA) that selectively assembled to one interface of the Janus emulsions and thus allowed us to tune the wettability profile of the resulting Janus particles upon UV-induced radical polymerization of the complex emulsion molds. Resulting Janus particles were accessible for a side-selective postfunctionalization reaction and as powerful solid surfactants for the stabilization of heterophasic air/water systems. As such, particles readily increased the content of dispersed air inside an aqueous continuous phase. Building upon the increased volume of dispersed gas, and as a novel application of Janus particles, we investigated their ability to increase the performance of an aqueous phase catalytic oxidation reaction. Attributed to the significantly increased availability of oxygen in proximity to catalytic reaction sites, we observed a 2.2-fold increase in the reaction rate, suggesting that stabilization of gas content inside a liquid phase using stable polymeric Janus particle surfactants, which are easily generated in one step, can provide a new tool to rationally enhance the general performance of heterophasic catalytic transformations in the future.

## Supporting Information Available

Details on synthetic procedures, contact angle measurements, calculation of droplet contact angle, volume ratio, and Janus ratio (PDF)
