# Supplementary material for: Synthesis of Polymer Janus Particles with Tunable Wettability Profiles as Potent Solid Surfactants to Promote Gas Delivery in Aqueous Reaction Media
Source: ACS Appl Mater Interfaces. 2021 Jun 29;13(27):32510–9. doi: 10.1021/acsami.1c07259 (PMC8283753; doi:10.1021/acsami.1c07259)
Supplement: Supplementary file 1 — am1c07259_si_001.pdf [file am1c07259_si_001.pdf]

Supporting Information

Synthesis of polymer Janus particles with tunable wettability profiles as potent solid surfactants to promote gas delivery in aqueous reaction media

*Bradley D. Frank,[1] Milena Perovic,[1] Saveh Djalali,[1] Markus Antonietti,[1] Martin Oschatz,[1,2] and Lukas Zeininger[1,]**

[1] Department of Colloid Chemistry, Max Planck Institute of Colloids and Interfaces, Am Mühlenberg 1, 14476 Potsdam, Germany; email: lukas.zeininger@mpikg.mpg.de

[2] Faculty of Chemistry and Earth Sciences, Friedrich-Schiller-University of Jena. Philosophenweg 7a, 07743 Jena, Germany.

Table of Contents

1. Synthesis of 10-(acryloyloxy)decanoic acid 3
2. Static contact angle measurement of particle surface analogues 5
3. Stability and phase separation of Janus droplet templates 6
4. Influence of ADA on particle morphology and fluorescence of covalently attached fluorescent marker 7
5. Particle morphology with continuous phase control 8
6. EDX and DLS of micron-scale Janus Particles 8
7. SEM of particles utilize for size-dependent oxygen delivery experiments 9
8. Calculation of droplet contact angle, volume ratio, and Janus ratio 10
9. Interfacial self-assembly and contact angle 12
10. Self-assembly of solid surfactant for gas stabilization 13
11. Interfacial behavior of synthesized Janus particles versus single-phase components 14
12. Self-assembly behavior of Janus particles versus single-phase counterparts 15

13. Janus particle bubble size versus solid-surfactant diameter 16
14. Supporting experiments for solid-surfactant mediated oxygen delivery 17
15. Synthesis of gold nanoparticles 17
16. NMR of reaction mixture post reaction 18
17. References 18

1. Synthesis of 10-(acryloyloxy)decanoic acid

10-(acryloyloxy) decanoic acid (ADA) was synthesized according to a modified literature procedure.[1] In brief, a solution of 10-hydroxydecanoic acid (1 g, 5.31 mmol) and trimethylamine (3 ml, 21.24 mmol) in 10 ml CH_2Cl_2 was added dropwise over 3 hours to a solution of acryloyl chloride (1.72 ml, 21.24 mmol) in 5 ml CH_2Cl_2 at -5 °C. Afterwards the mixture was stirred overnight at 0 °C. 2 ml of water was added, and then a solution of NaOH (1.13 g, 28.25 mmol) in 2.5 ml water was added dropwise at -5 °C. At this temperature the mixture was stirred for 45 min. The reaction solution was acidified in the cold with conc. HCl-solution to pH 1. The solvent was removed by rotary-evaporation under reduced pressure. The product was received as a water-insoluble yellow liquid. Scheme, yield, and NMR can be found.

Reaction scheme:

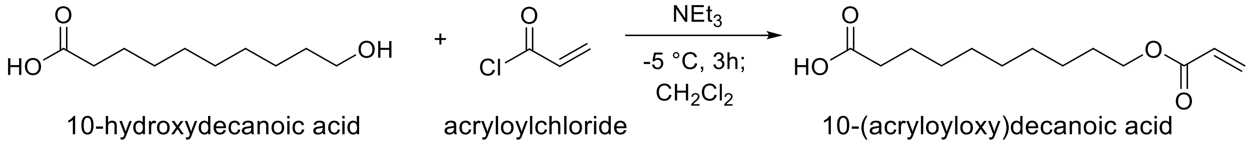

Yield: 1.675 g (75 %)

^{1}H-NMR (400 MHz, $CDCl_3$): 1.29 (m, 10 H), 1.62 (m, 4 H), 2.33 (t, 2H), 4.13 (t, 2H), 5.76-6.50 (m, 3H), 8.15 (s, 1H).

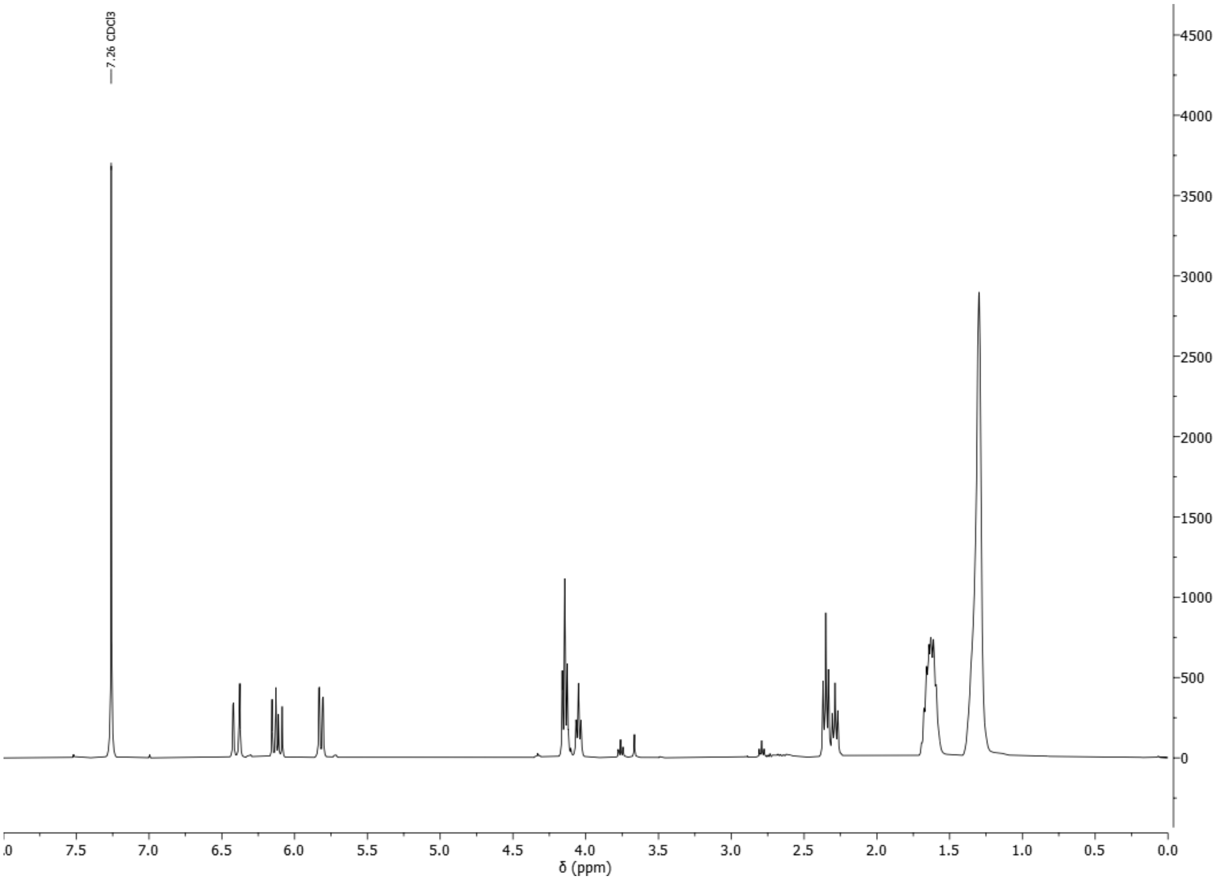

Figure S1. ^{1}H-NMR Spectra of 10-(acryloyloxy)decanoic acid.

2. Static contact angle measurement of particle surface analogues

Estimation of the individual Janus particle phase hydrophilicity, and thus its performance as a Pickering emulsifier was conducted via the production of polymer surfaces in like-conditions to the polymer particle. For this, monomer of the matching phase was drop casted into the like-condition of the polymer particle, polymerized with UV light, and static contact angle measured with respect to water. For example, to measure a HDDA surface with 9 mg/mL ADA, 0.2 mL of the monomer solution containing 4 % D1173 and 9 mg/mL ADA in HDDA was prepared and drop casted into a surfactant solution containing 4 mL of 7:3 WT % SDS : Zonyl contained in a polystyrene petri dish. This film was then polymerized with UV light, dried, and cleaned with water and methanol before observation of the static contact angle. A table of the relevant static contact angle analogues is listed in Table S1.

Table S1. Static contact angle of water on the polymer surface analogues relevant to particles presented in this paper, displaying internal-surfactant driven contact angle change.

Polymer Substrate	Functionality	Static Contact Angle of Water
HDDA	None	76° ± 1
HDDA	9 mg/mL AODA	56° ± 1
PFDA	None	120° ± 3

3. Stability and phase separation of Janus droplet templates

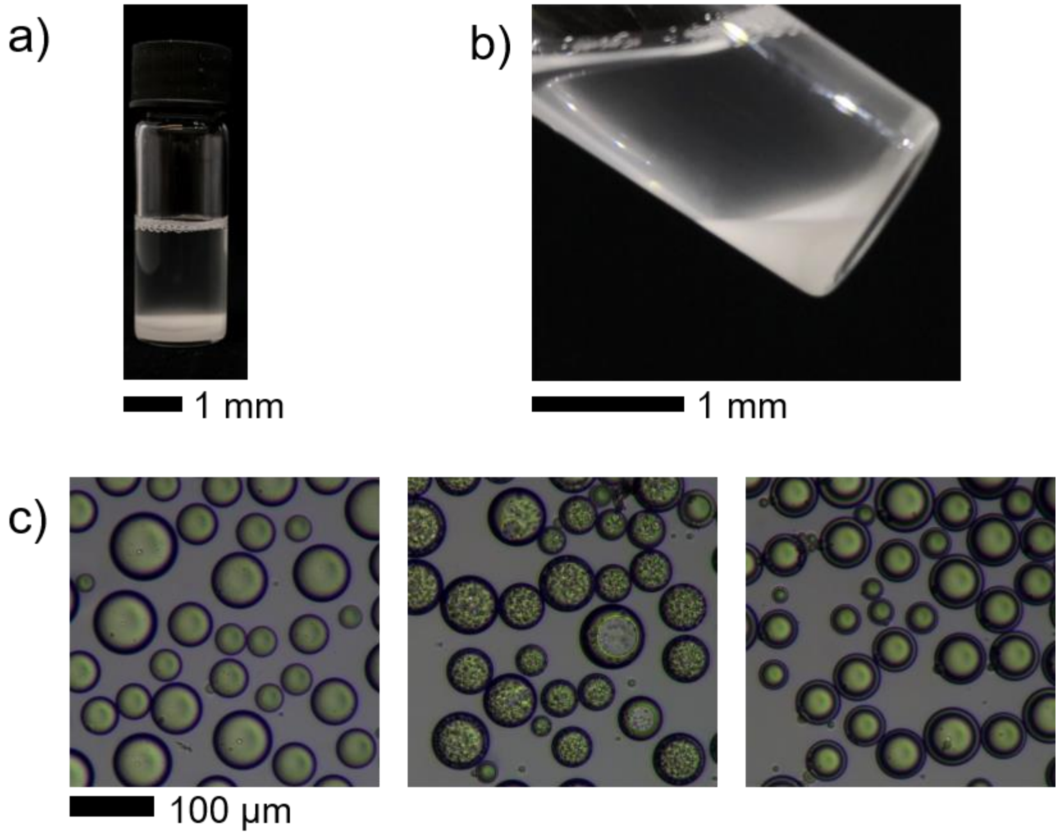

Figure S3. (**a**) Photograph of Janus emulsions comprised of a solution of 6 mg mL^{-1} ADA in 1,6 hexanediol diacrylate with 5 wt % trimethylolpropane ethoxylate triacrylate and 5 wt % darocur 1173 as the hydrocarbon phase (HC) and perfluorodecyl acrylate as the fluorocarbon phase (FC) in a volume ratio of 1:1. (**b**) Photograph of the non-phase separated vial tilted to show the lack of de-emulsification present at the vial bottom. (**d**) Inverted optical micrographs of non-phase separated emulsion droplets, and the phase separation progress (left to right) over 1 hour.

4. Influence of ADA on particle morphology and fluorescence of covalently attached fluorescent marker

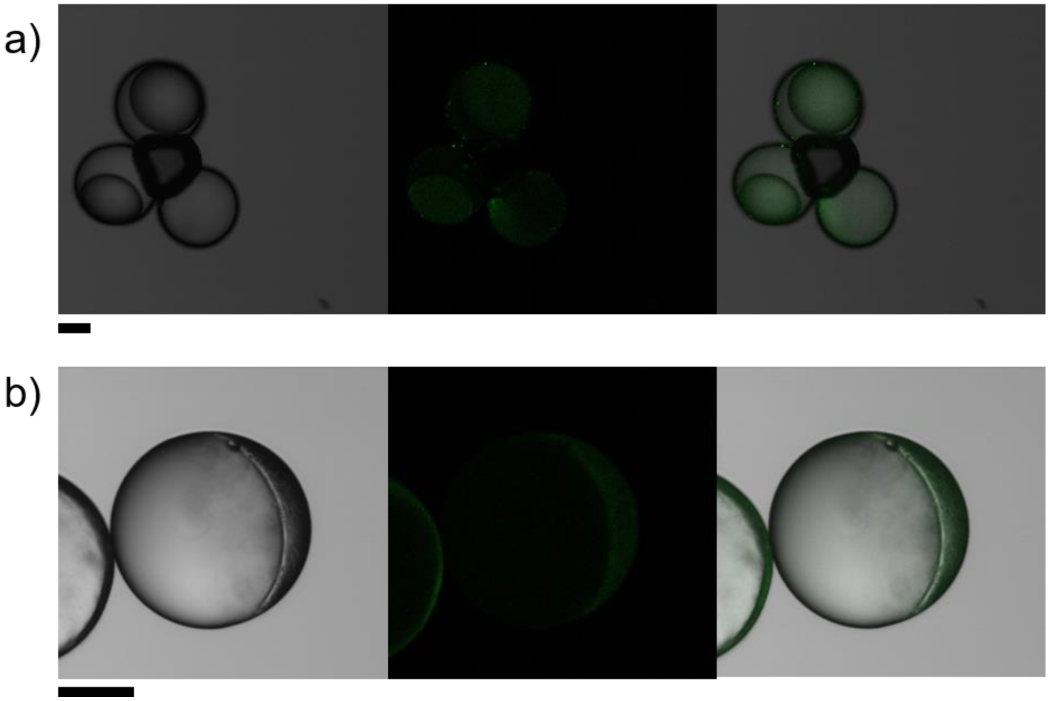

Figure S4. (**a**) Transmitted light detector (TLD), fluorescence, and overlaid images of particles produced with 2 mg/mL ADA (HC phase) with 1:1 HDDA : PFDA with 5 WT % D1173, in 7:3 1 WT% SDS:Zonyl, after EDC coupling with fluoresceinamine. (**b**) TLD, fluorescence, and overlaid images of particles generated with 6 mg/mL ADA (HC phase) with 1:1 HC : FC monomer HDDA : PFDA with 5 WT % D1173, in 7:3 1 WT % SDS:Zonyl, scale bar = 50µm.

5. Particle morphology with continuous phase control

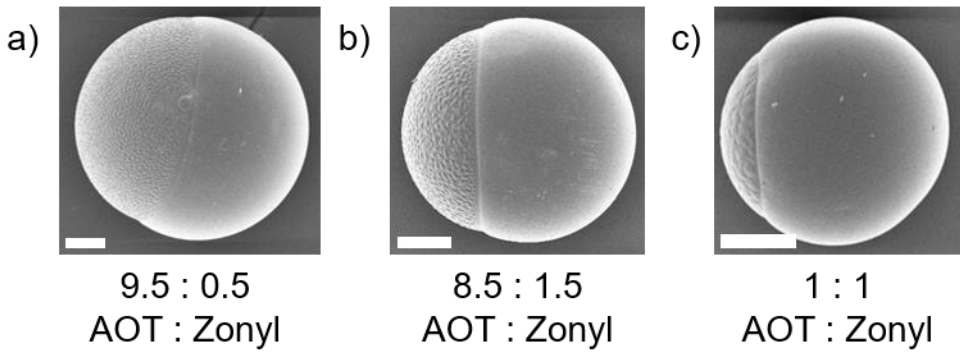

Figure S5. SEM of particle morphology with surfactant based control. 1:1 mixture of 6mg/mL ADA in 1,6 HDDA : PFDA w/ 5 WT% D1173, in different solutions of 1 WT% AOT : Zonyl as noted in the image. With increasing Zonyl, the fluorocarbon phase portion increases from A to C, scale bar = 20μm.

6. EDX and DLS of micron-scale Janus Particles

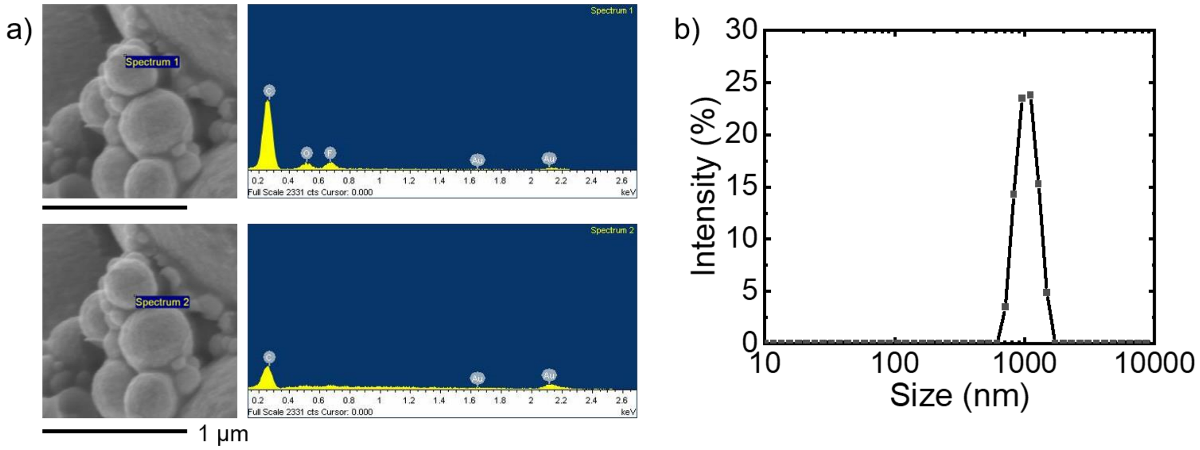

Figure S6. (**a**) Inset SEM with EDX point spectra from either hemisphere of a <1μm Janus particle displaying the phase separation, as a function of the carbon content and fluorocarbon content of the opposing precursors. (**b**) DLS of Janus particle size distribution for the 1 μm particles used in the work.

7. SEM of particles utilize for size-dependent oxygen delivery experiments

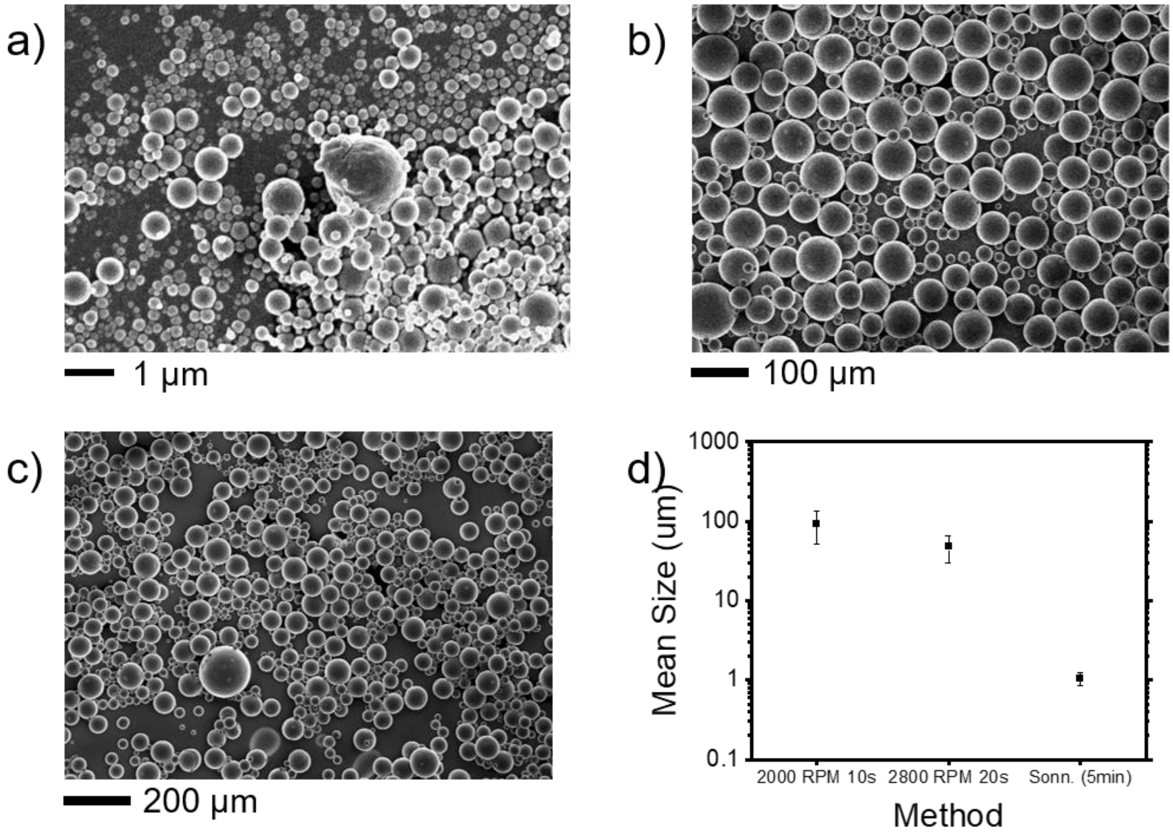

Figure S7. Scanning electron micrographs of various particles used at different. (**a**) 1 μm particles, as featured in Figure S6 synthesized with sonication methods (180W; 45kHz; 5 min). (**b**) 50 μm particles synthesized with vortex mixing (20s at 2800 RPM). (**c**) 100 μm particles synthesized with vortex mixing (10s at 2500 RPM). (**d**) Comparative mean size and distribution of particles used in this method, measured with a combination of optical methods and DLS.

8. Calculation of droplet contact angle, volume ratio, and Janus ratio

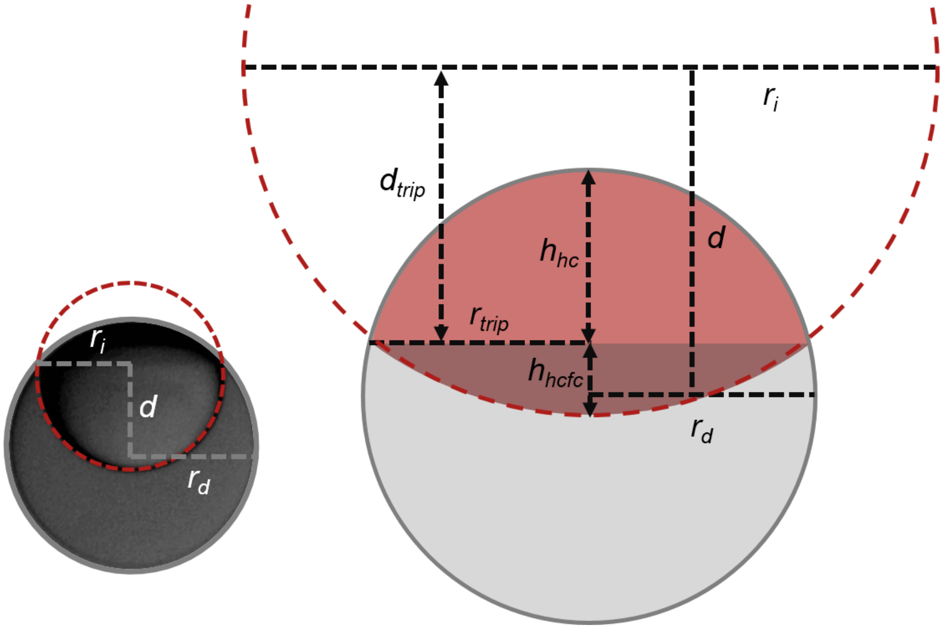

Figure S8. Scheme for the calculation of droplet contact angle, volume ratio, and Janus ratio from a complex emulsion or particle, from the measurements of the internal radius, droplet radius, and distance between two circles of a complex droplet imaged with a sideview optical microscope.

Given low interfacial contact angles between two oil phases for a complex droplet, the morphology of the droplet is defined by a balance of interfacial tensions, and the geometry of complex droplets have been well defined.[1] For our droplets and resulting particles, due to essentially retained morphology on polymerization, side view optical micrographs allow us to study. As a result, the droplet contact angle, volume ratio, and Janus ratio of the particles can all be calculated from the radii of two circles, as well as the distance between the two circles. The two circles are defined as the entire droplet, and a circle defined by the internal curvature of the HC/FC interface. For the triple-phase contact angle ϕ, this can be calculated as:

$$\phi = \cos^{-1}\left(\frac{r_i^2 + r_d^2 - d^2}{2 r_d r_i}\right) \tag{1}$$

for ϕ between 0 and 90 degrees measured from two circles and the distance between their centerpoints. The height of either cap can either be measured from sideview optical micrographs with Fiji or similar software, or calculated with the either radii and the distance of the triple phase boundary:

$$d_{trip} = (d^2 - r_d^2 + r_i^2)/2d \quad (2)$$

With the distance to the triple phase boundary, the height of the cap h_{hcfc} or h_{hc} is calculated from (2):

$$h_{hcfc} = r_i - d_{trip} \text{ and } h_{hc} = r_d - d - d_{trip} \quad (3)$$

For the volume ratio of droplets, for the relation between the volume ratio, Janus ratio, and contact angle, volume ratio is calculated as:

$$V_{HCFC} = V_{HC}/\ V_{FC} \quad (4)$$

where the volume of the HC is the volume of the two spherical caps defined by the radius of the droplet or internal curvature and the height of the corresponding cap from (3):

$$V_{cap} = \frac{\pi}{3} h^2(3r - h) \quad (5)$$

With the volume of the hydrocarbon phase, the volume of the fluorocarbon phase is calculated with (5) and the volume of a sphere as:

$$V_{FC} = V_{droplet} - V_{HC} + V_{HCFC} \quad (6)$$

From this, V_{cap} is obtained for both the cap defined by the droplet curvature and by the internal curvature, and the ratio of volumes can be obtained. Particle Janus ratio, or the ratio of surface areas of particle faces, is then defined by the surface area of the exposed HC cap calculated from (3):

$$A_c = 2\pi r_d h_{cap} \quad (7)$$

The surface area of the FC cap is then the surface area of the spherical droplet without the HC cap:

$$A_{FC} = A_{drop} - A_{HC} \quad (8)$$

and the surface area of the hydrocarbon portion is calculated from the radius of the particle or droplet, and the height of the cap:

$$A_{HCFC} = A_{HC}/A_{FC} \quad (9)$$

9. Interfacial self-assembly and contact angle

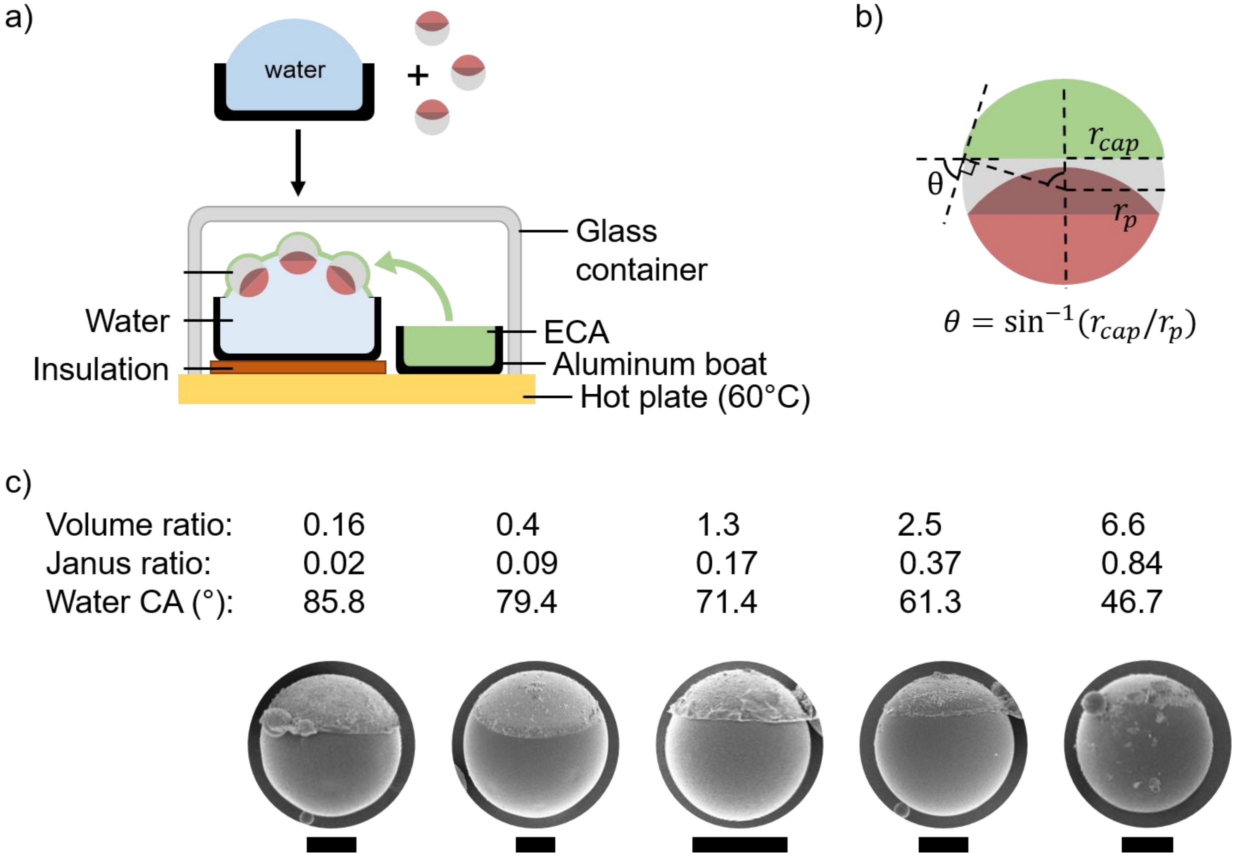

Figure S9. (**a**) Expanded schematic of the particle ECA treatment method for the measurement of interfacial contact angle of complex particles. (**b**) Demonstrative schematic for the calculation of interfacial calculation from ECA treatment. (**c**) Table of particle volume ratio (V_{HC}/V_{FC}), Janus ratio (A_{HC}/A_{FC}), and water contact angle (°) as calculated from ECA treatment, with scanning electron micrographs of ECA-treated Janus particles for the determination of interfacial contact angles, generated from Janus emulsions with varied volume ratios of monomer precursors, scale bar = 20μm.

10. Self-assembly of solid surfactant for gas stabilization

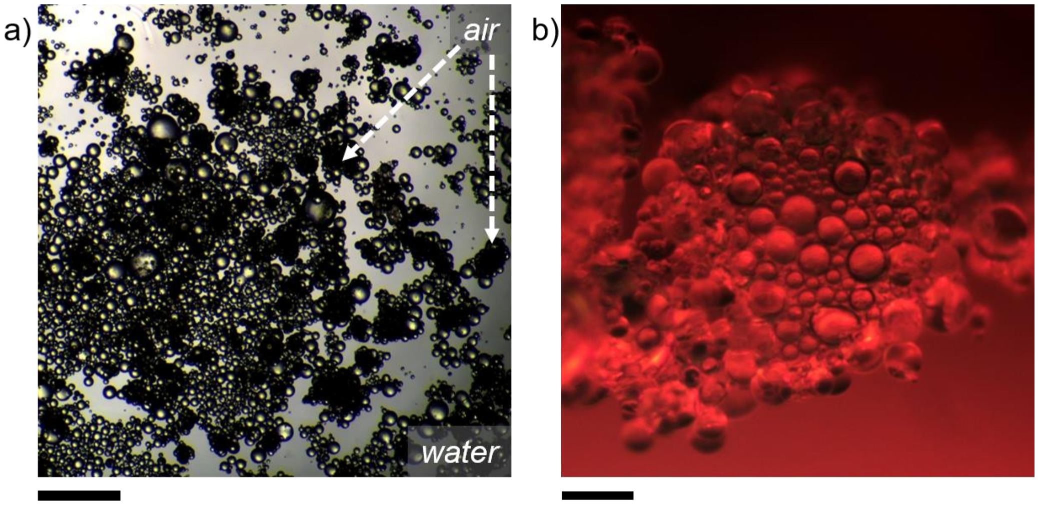

Figure S10. (**a**) Inverted optical micrographs of presented solid surfactant self-assembly via forced immersion and hydrophobic interactions, trapping air (black due to refractive index contrast with water) in solution, scale bar = 500µm. (**b**) Sideview optical micrograph of presented solid surfactant stabilizing air-in-AuNP solution used in catalysis reactions, where solid surfactant is stabilizing the air-water interface in solution, scale bar = 100µm.

11. Interfacial behavior of synthesized Janus particles versus single-phase components

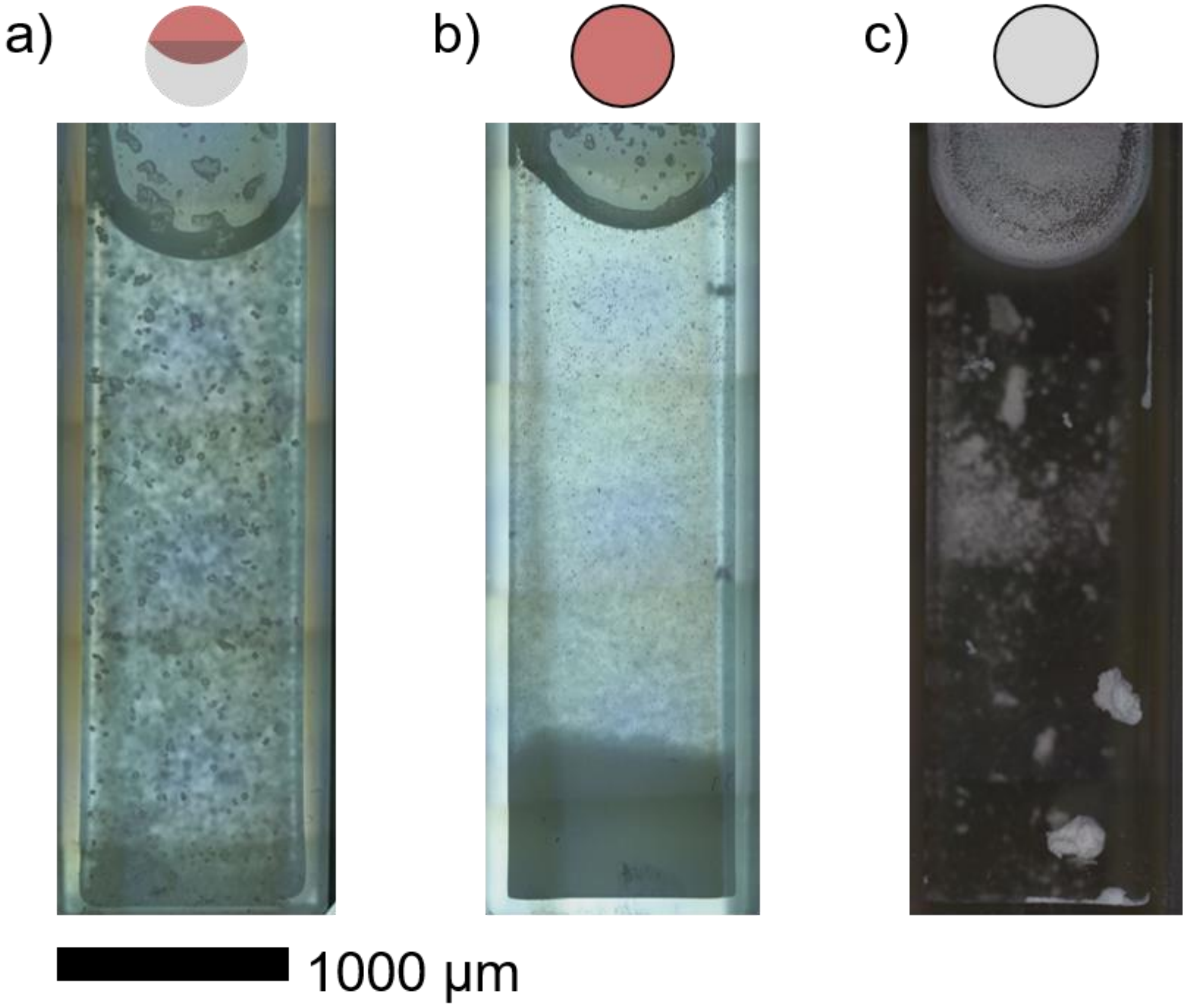

Figure S11. (**a**) Horizontally placed full-vial stitched optical micrographs of the synthesized Janus particles (10 mgmL^{-1}) with an interfacial contact angle of $\theta = 61°$ (50µm diameter, Figure 4a), as well as particles made of purely (**b**) 1,6 hexanediol diacrylate with 6 mgmL^{-1} ADA, and (**c**) perfluorodecyl acrylate particles. All vials were vortex mixed for 20 seconds at 2500 RPM before imaging with a vertical microscope, showing the bubble formation of the Janus particles in (a), the dispersibility of the hydrophilic single phase particles in (b), and the non-dispersibility of the very hydrophobic single phase particles in (c), where some self-assembly occurs.

12. Self-assembly behavior of Janus particles versus single-phase counterparts

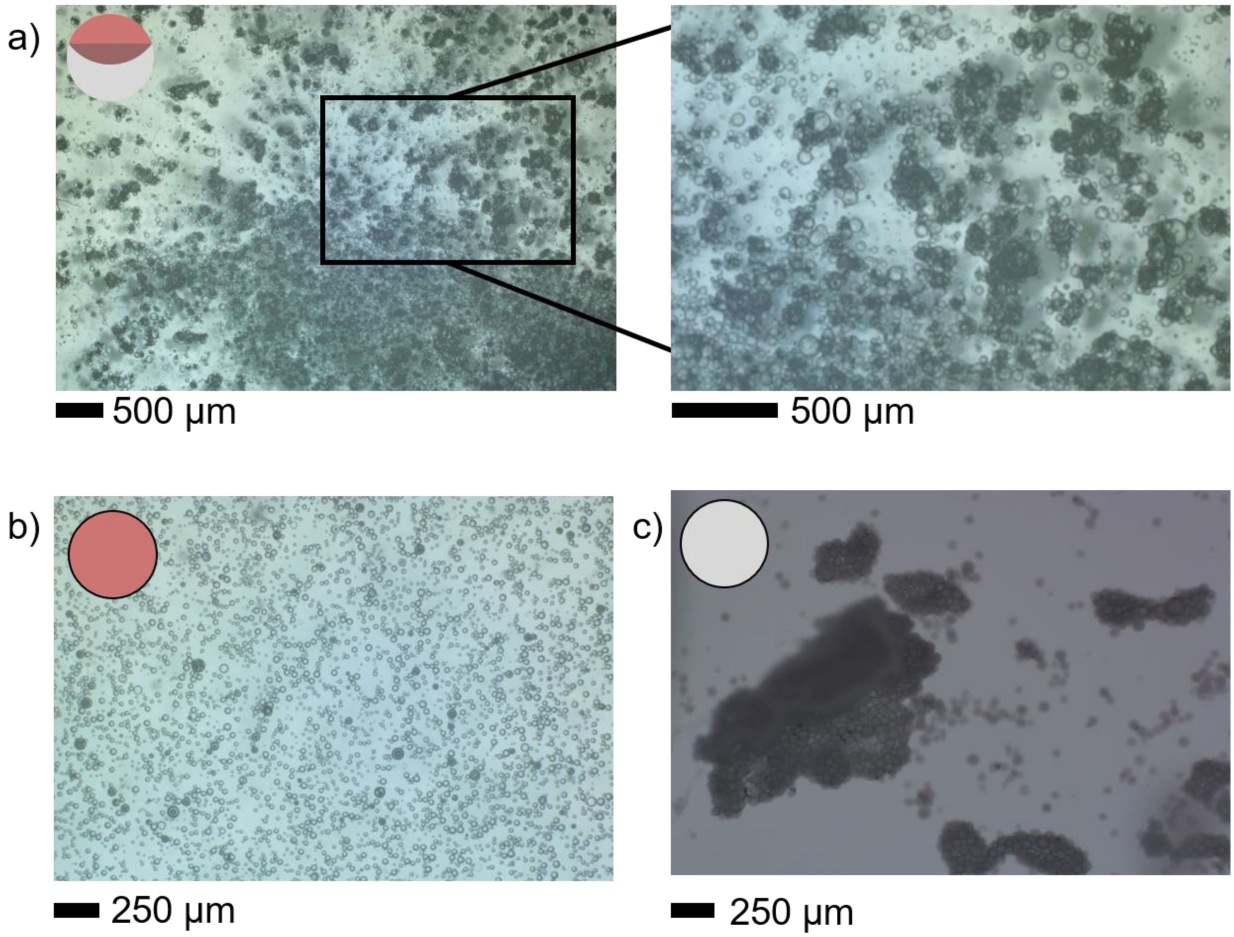

Figure S12. (**a**) Optical micrographs of the bottom of the bottom of the vial in Figure S9a, where the micro-assembly of gas-containing Janus particles (50µm diameter, interfacial contact angle of $\theta = 61°$, Figure 4a) can be observed. (**b**) Optical micrograph of single-phase hydrophilic particles composed 1,6 hexanediol diacrylate with 6 mgmL^{-1} ADA, where the particles are dispersible in water, as they sit in Figure S9b at the bottom of the vial. (**c**) Optical micrograph of single-phase hydrophobic particles composed of perfluorodecyl acrylate, where the particles are largely not dispersible in water, and assemble into large structures.

13. Janus particle bubble size versus solid-surfactant diameter

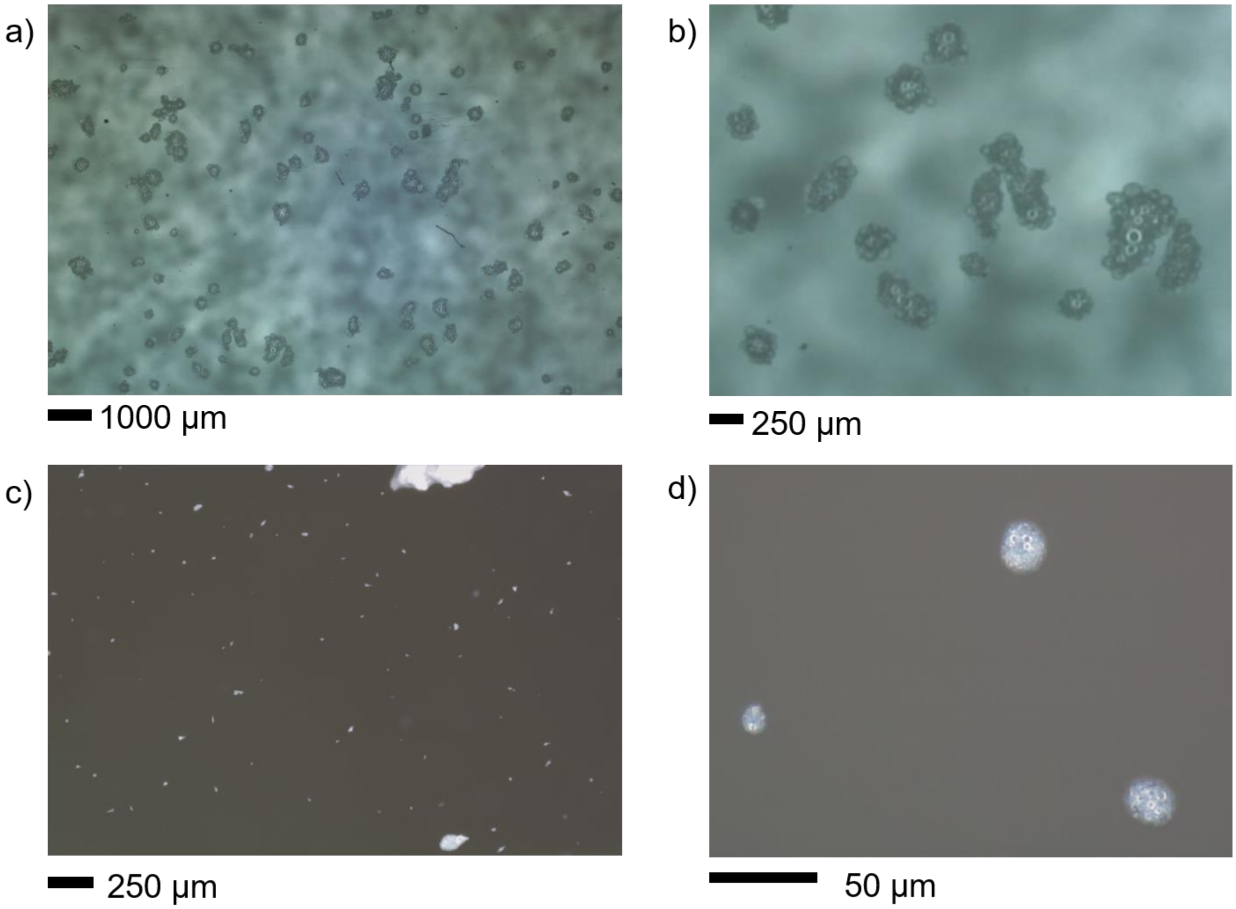

Figure S13. (**a-b**) Optical micrographs of the top of the cuvette from Figure S11a, where the solid-surfactant used (50µm diameter, interfacial contact angle of $\theta = 61°$, Figure 4a) is stabilizing floating air-in-water bubbles with a mean diameter of 301 ± 332 µm. (**c-d**) Optical micrographs of the top of a cuvette containing 1µm diameter Janus particles stabilizing floating air-in-water bubbles with a mean diameter of 23 ± 14 µm.

14. Supporting experiments for solid-surfactant mediated oxygen delivery

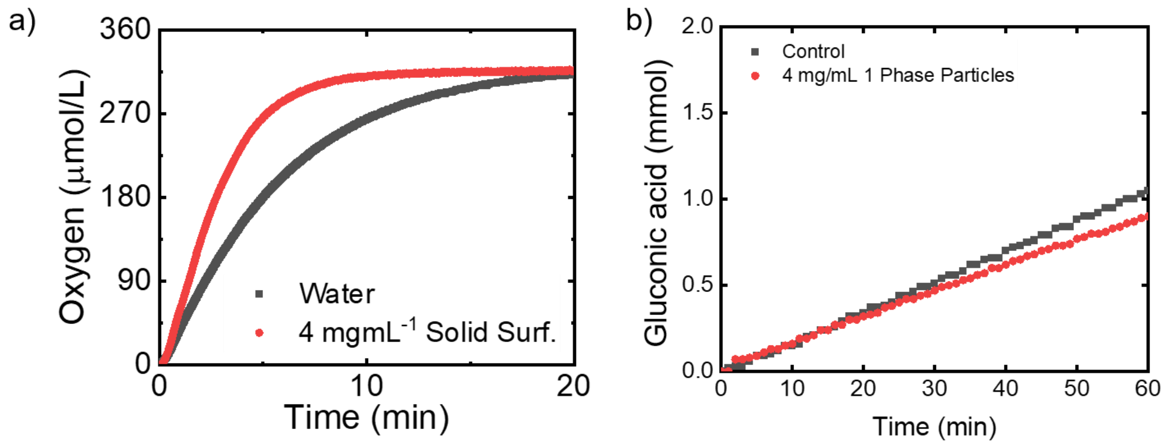

Figure S14. (**a**) Dissolved oxygen content in water over time of a flask with 50 mL of deoxygenated water (nitrogen bubbling) after exposure to ambient air with and without the addition of 4 $mgmL^{-1}$ solid surfactant (50µm diameter, interfacial contact angle of $\theta = 61°$, Figure 4a). (**c**) Reaction monitoring via titration of 1M sodium hydroxide for the gold-mediated conversion of D-glucose to gluconic acid with and without 4 $mgmL^{-1}$ single-phase polymer particles, composed of the same polymers used in this study without phase de-mixing (reaction conditions: 10 mL min^{-1} O_2, 0.4 M glucose, 7.36 10^{-5} $mol_{Au}mol_{glc}^{-1}$ Au nanoparticles, stirring: 1000 RPM, T = 30°C).

15. Synthesis of gold nanoparticles

The gold nanoparticle catalyst was synthesized according to a literature-known procedure.[2] In brief, we added 29 mL of 0.2 wt % gold(III) chloride trihydrate to 500 mL of water under stirring. Subsequently, 1 wt % sodium citrate was added (11.6 mL), before 5.80 mL of sodium borohydride solution (1.7 mg mL^{-1} sodium borohydride and 10 mg mL^{-1} sodium citrate).

16. NMR of reaction mixture post reaction

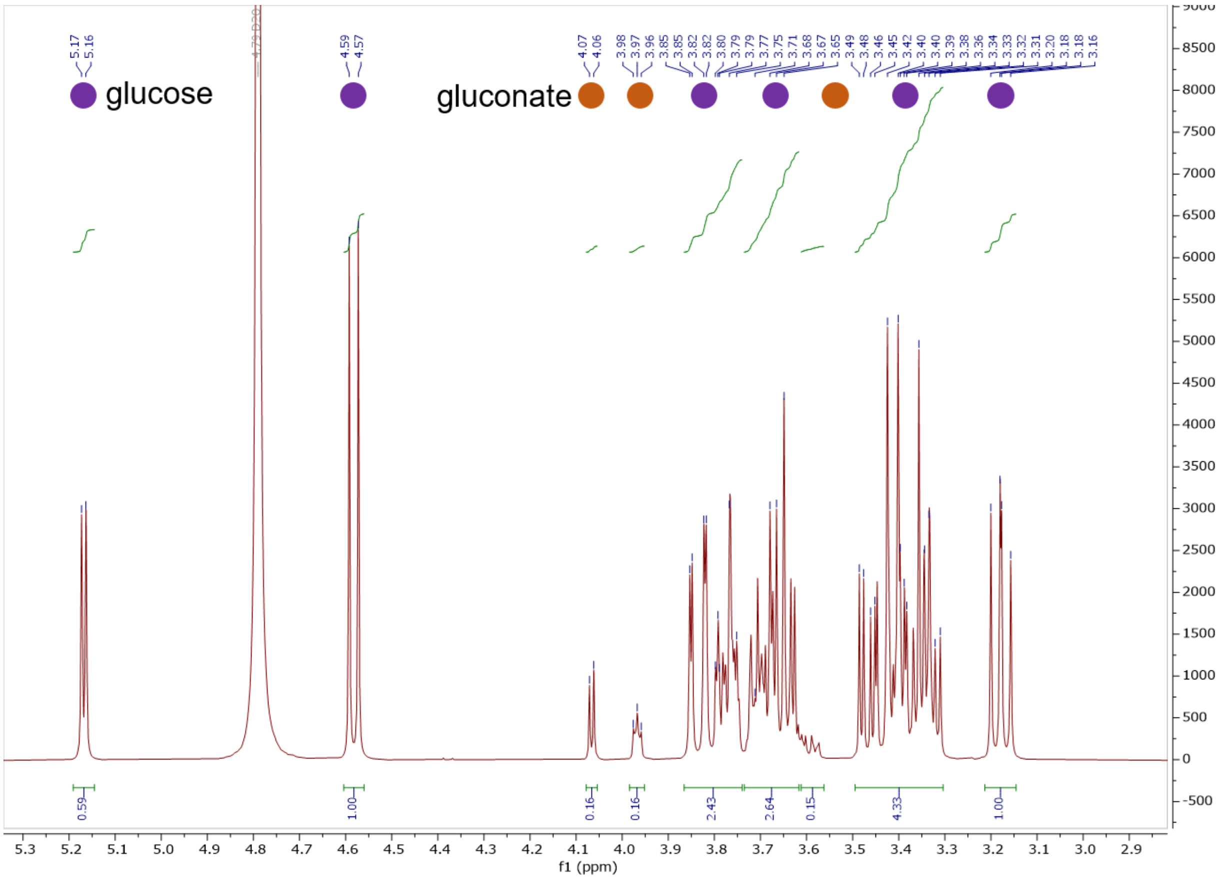

Figure S15. ^{1}H-NMR Spectra of reaction mixture after 3h with AuNPs, Janus Particles, (400 MHz, D_2O) δ 4.07 (d, J = 3.8 Hz, 1H), 3.97 (dd, 1H), 3.62 –3.58 (m, 1H).

17. References

(1) Song, C.; An, Huiyong.; Yang, Q.; Li, W.; Liu, C.; Wang, P. Synthesis, characterization, and aqueous solution behavior of copolymers of acrylamide and sodium 10-acryloyloxydecanoate. *Polym. Bull*. **2011**, 67(9):1917–1934.

(2) Turkevich, J.; Stevenson, P. C.; Hillier, J. A study of the nucleation and growth processes in the synthesis of colloidal gold. Discuss. Faraday Soc. **1951**, 11, 55-75.
